# Characterization and identification of hidden rare variants in the human genome

**DOI:** 10.1186/s12864-015-1481-9

**Published:** 2015-04-24

**Authors:** Alberto Magi, Romina D’Aurizio, Flavia Palombo, Ingrid Cifola, Lorenzo Tattini, Roberto Semeraro, Tommaso Pippucci, Betti Giusti, Giovanni Romeo, Rosanna Abbate, Gian Franco Gensini

**Affiliations:** Department of Experimental and Clinical Medicine, University of Florence, Florence, Italy; Laboratory of Integrative Systems Medicine (LISM), Institute of Informatics and Telematics and Institute of Clinical Physiology, National Research Council, Pisa, Italy; Medical Genetics Unit, Department of Medical and Surgical Sciences, University of Bologna, Bologna, Italy; Institute for Biomedical Technologies, National Research Council, Milan, Italy; Department of Neuroscience, Pharmacology and Child Health, University of Florence, Florence, Italy

## Abstract

**Background:**

By examining the genotype calls generated by the 1000 Genomes Project we discovered that the human reference genome GRCh37 contains almost 20,000 loci in which the reference allele has never been observed in healthy individuals and around 70,000 loci in which it has been observed only in the heterozygous state.

**Results:**

We show that a large fraction of this rare reference allele (RRA) loci belongs to coding, functional and regulatory elements of the genome and could be linked to rare Mendelian disorders as well as cancer. We also demonstrate that classical germline and somatic variant calling tools are not capable to recognize the rare allele when present in these loci. To overcome such limitations, we developed a novel tool, named RAREVATOR, that is able to identify and call the rare allele in these genomic positions. By using a small cancer dataset we compared our tool with two state-of-the-art callers and we found that RAREVATOR identified more than 1,500 germline and 22 somatic RRA variants missed by the two methods and which belong to significantly mutated pathways.

**Conclusions:**

These results show that, to date, the investigation of around 100,000 loci of the human genome has been missed by re-sequencing experiments based on the GRCh37 assembly and that our tool can fill the gap left by other methods. Moreover, the investigation of the latest version of the human reference genome, GRCh38, showed that although the GRC corrected almost all insertions and a small part of SNVs and deletions, a large number of functionally relevant RRAs still remain unchanged. For this reason, also future resequencing experiments, based on GRCh38, will benefit from RAREVATOR analysis results. RAREVATOR is freely available at http://sourceforge.net/projects/rarevator.

**Electronic supplementary material:**

The online version of this article (doi:10.1186/s12864-015-1481-9) contains supplementary material, which is available to authorized users.

## Background

Thanks to novel high-throughput sequencing (HTS) technologies [1-3], today a human genome can be sequenced very quickly at affordable prices. The emergence of these platforms, together with the development of powerful computational tools, have transformed biological and biomedical research over the past several years allowing the achievement of large-scale population sequencing projects, such as the 1000 Genomes Project (1000GP) [4] and The Cancer Genome Atlas (www.cancergenome.nih.gov), and opened a new era for personal genomics [5-7]. Existing HTS technologies generate billions of short sequences (reads of ∼ 100-450 base pairs), and although computational methods may permit routine use of *de novo* assembly, sequencing a human genome typically allows to relate sequence information to a reference haploid genome: the so-called resequencing strategy.

In re-sequencing approach, the key first step is the alignment, or mapping, of all the reads to a reference genome by using short read alignment tools [8,9]. Once the reads have been properly mapped, genomic variants can be discovered by identifying differences between the reference genome and the aligned reads. By using this procedure, it is possible to identify single nucleotide variants (SNVs) [10,11], small insertions and deletions (InDels) [12] and infer DNA copy number variants [13,14].

In diploid genomes, such as the human genome, variants can be found in one chromosome (heterozygous) or in both chromosomes (homozygous). In the first case, the variant can be responsible for dominant phenotypes, while in the latter for recessive phenotypes. The identification of homozygous and heterozygous variants have a strong medical relevance since it is at the base of the discovery of loss-of-function and gain-of-function mutations. Loss-of-function variants in homozygous state are the most common cause of autosomal recessive Mendelian disorders, while gain-of-function variants that change the gene product to a new and abnormal function usually lead to dominant Mendelian disorders. Moreover, both loss-of-function and gain-of-function mutations inherited in germline cells or acquired in somatic cells are often the starting events of cancer evolution and proliferation. Functionally relevant variants responsible for mendelian disorders [15] and cancer [16] have been successfully identified by using the re-sequencing strategy [15,16].

The discovery of a functionally relevant variant requires to identify differences between aligned reads and the sequence of the haploid reference genome. The sequence of the human reference genome [17] was obtained from a collection of DNAs from anonymous individuals with primarily European origins and assembled into a mosaic haploid genome. The clinical and phenotypic information of the participants is unknown. Although they were likely to be healthy at the time of study, some of them might be carriers of disease risk alleles. The human reference genome is maintained and updated by the Genome Reference Consortium (GRC) which is responsible to correct the small number of regions in the reference that are currently misrepresented, to close as many remaining gaps as possible and to produce alternative assemblies of structurally variant loci when necessary. Since 2009, the major assembly release for human genome has been GRCh37 that is present in various genome browsers and databases including Ensembl, NCBI and UCSC Genome Browser. In December 2013, the GRC announced the public release of GRCh38, the latest version of the human reference genome assembly. This represents the first major assembly update since 2009, and introduces changes to chromosome coordinates.

Large scale population re-sequencing projects, such as the 1000GP and the Exome Sequencing Project (ESP), allowed to create a large and detailed catalogue of human genetic variations and improved our knowledge of the human genetic variation. Recently, the 1000GP Consortium, by combining low-coverage whole-genome sequencing (WGS) and high-coverage whole-exome sequencing (WES) of 1092 individuals from 14 populations, identified around 38 million single nucleotide polymorphic positions and 1.4 million short insertions and deletions [18].

In this work, we re-analyzed the complete set of these variant calls, and we found that more than 96000 loci of the GRCh37 assembly have a reference allele frequency smaller than 1% (here called Rare Reference Alleles, RRAs). Moreover, we also discovered that for the great majority of these loci the reference allele has never been observed in homozygous state and for a significant part neither in heterozygous state. These findings suggest that for many of these loci the reference allele could have functional consequences or even being a loss/gain-of-function variant. To evaluate the biological relevance and the deleteriousness of finding the reference allele in these loci we studied them by using several annotation databases and measures of functional impact. We found that a large fraction of these loci belong to genomic features identified by GENCODE [19] or regulatory elements discovered by the ENCODE project [20], and a significant fraction has functional consequences on genes that are linked to Mendelian disorders, complex diseases and cancer. We also demonstrated that currently available computational approaches for the detection of germline and somatic SNVs and InDels fail to call variants in RRA loci when one of the alleles is the reference allele. To complement the variant calls from currently available tools we developed a novel software package for the detection and annotation of germline and somatic variants in RRA loci that we named RAREVATOR (RAre REference VAriant annotaTOR). As a proof of principle, we applied RAREVATOR to the analysis of a publicly available whole-exome sequencing cancer dataset and we compared these results with those obtained by two state-of-the-art variant callers, VarScan2 [21] and Mutect [22]. Our tool was able to identify around 1500 germline and 22 somatic RRA variants that were missed by the other two tools. We further searched for significantly mutated genes and pathways and showed that some of these RRA variants belong to signicantly mutated pathways and can thus have a role in human development and tumorigenesis.

## Results

### Variant analysis

We downloaded the complete set of variant calls produced by the 1000GP Consortium from ftp://ftp-trace.ncbi.nih.gov/1000genomes/ftp/release/20110521 and we estimated the allele frequency of all the SNVs and small InDels across the 1092 individuals (see Methods for more details). We found that 85,111 SNV loci and 11,162 small InDel loci (6,700 Insertions and 4,462 Deletions) have a reference allele frequency (AF) smaller than 0.01, revealing that almost 100,000 loci of the GRCh37 assembly are RRA loci (Table 1).

**Table 1:** **Summary statistics of the RRA loci**

**Variants**	**Total number**	**Class A**	**Class B**	**Class C**	**Total ESP**	**ESP AF ≤ 0.01**	**ESP AF ≤ 0.03**
SNV	85111	17944	60714	6453	1033	862	1033
InDels	11162	3076	7711	375	170	152	169

Columns report the total number of variants (Total Number), the number of variants that belong to classes A, B and C, the number of RRA loci studied by ESP (Total ESP), the number of RRA loci with allele frequency calculated by ESP smaller than 0.01 (ESP AF ≤ 0.01) and 0.03 (ESP AF ≤ 0.03).

To evaluate the reliability of these results, we compared the allele frequencies estimated by the 1000GP Consortium with those calculated by the NHLBI GO Exome Sequencing Project (ESP) on the same loci (http://evs.gs.washington.edu/EVS). Since the ESP sequencing experiments have been performed for coding regions (see Methods for more details), only 1033 of the 85,111 SNVs and 170 of the 11162 InDel overlap with the ESP variants. Despite the limited number of shared variants, and the fact that ESP sequenced individuals affected by several disorders, we found strong concordance between the two datasets. All the 1033 SNVs have a reference AF < 0.05 and 862 SNVs < 0.01, while 152 of the 170 InDels have a reference AF < 0.01 (Table 1).

To investigate the genetic characteristics of these loci, we calculated their genotype counts (see Methods for more details) and we classified them into three classes. The first class (A) contains the loci in which the reference allele has never been observed in 1000GP individuals and for this reason it could have loss-of-function as well as gain-of-function role. In class B, the reference allele has been observed only in the heterozygous state and could consequently have a loss-of-fuction role. Finally, for class C loci, the reference allele has been observed in both heterozygous and homozygous state in at least one individual and for this reason the reference allele could have moderate phenotypic impact. The results of this analysis are summarized in Table 1 and show that 17,940 SNVs and 3,080 InDel loci (1870 insertions and 1210 deletions) are of class A, 60,711 SNVs and 7,715 InDel loci (4,616 insertions and 3,099 deletions) of class B and 6,460 SNVs and 369 InDels loci (214 Insertions and 155 deletions) of class C.

**Figure 1:**
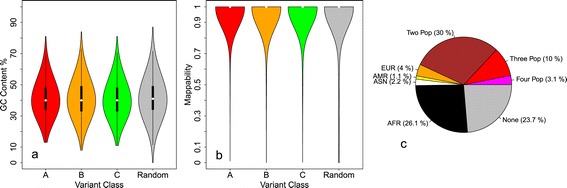
GC content, mappability and population distribution of RRA loci. Panels **a** and **b** report the distribution of GC-content percent and mappability of the 100 bp region surrounding each RRA locus (for RRA classes A, B and C) compared to randomly sampled 100 bp regions. Panel **c** report the distribution of RRA loci across macro populations: African (AFR), American (AMR), Asian (ASN), European (EUR), shared by none (None), two (Two Pop), three (Three Pop) and four populations (Four Pop).

To understand if these alleles are sequencing artifacts belonging to complex and hard-to-sequence regions of the genome, we calculated the GC content and mappability of the 100 bp region surrounding each RRA locus and we compared them with 100 bp regions randomly sampled from the genome. Although a small fraction of these regions have values of GC-content and mappability that could affect the correct identification of variant alleles, globally the distributions of GC content and mappability calculated for RRA loci (Figure 1a-b) are identical to the distributions of randomly sampled regions. These results suggest that the presence of RRA alleles in the human genome reference sequence can not be fully ascribable to issues of genome complexity or sequencing errors. As a further step, in order to understand if RRAs are rare/private variants of individuals used by the human genome project, we studied the presence of these alleles in the four different macro populations of the 1000GP: European (EUR), American (AMR), Asian (ASN) and African (AFR) (Figure 1c). Almost 45% of the RRAs are shared by two, three or all four macro populations, while more than 7% belong to individuals from European, American or Asian origins. Interestingly, more than 25% of the RRAs are private/rare variants of individuals from African populations, and this is in accordance with the fact that the majority of the reference DNA used by the human genome project was from donor RP11, an individual likely of African-American ancestry [23,24]. Overall, from these results we can conclude that the presence of these rare alleles in the reference assembly of the human genome can be ascribed to both sequencing errors and private variants of the anonymous individuals used by the human genome project.

### Variant annotation

#### GENCODE

SNVs and small InDels in protein-coding genes can severely affect the function of the encoded proteins and lead to disease states [25,26]. Exonic SNVs such as missense mutations resulting in amino acid substitutions and nonsense mutations introducing new stop codons can seriously disrupt the function of human protein-coding genes. Frameshifting InDels result in a completely different translation of the original protein that could be abnormally shorter or longer, likely altering its function. Variants in intronic regions affecting constitutive splice sites may have severe functional consequences similar to nonsense or missense mutations, resulting in aberrantly included introns or skipped exons, and sometimes in nonsense-mediated decay. Moreover, in the last few years, it has become clear that also the non-protein-coding portion of the genome is of crucial importance for normal development and physiology and for and for the appearance of disease phenotypes. This is particularly evident for microRNAs for which it has been shown that genetic changes are hallmark of cancers, but also variants in large intergenic non-coding RNAs (lincRNAs), small nucleolar RNAs (snoRNAs) and other ncRNAs might contribute to the development of many different human disorders [27]. To understand the functional relevance of all the RRA loci we studied their overlap with the biotypes annotated by the GENCODE consortium (see Methods for more details) and we found that more than half (44790 SNVs, 3788 Insertions and 2,593 Deletions, see Table 2 and Additional file 1: Table S1) belong to GENCODE features. Specifically, 35,428 SNVs and 5,060 InDels (2983 insertions and 2,077 deletions) are part of protein-coding genes, while 8,628 SNVs and 1,230 InDel (744 insertions and 486 deletions) belong to ncRNA features. The most represented non-coding biotype is lincRNA, followed by antisense and processed transcripts, while 734 SNVs and 91 InDels (61 insertions and 30 deletions) belong to pseudogenes. Genotype counts analysis show that around 25% of the GENCODE loci belongs to class A variants (9,940 SNVs, 1119 insertions and 702 deletions) and around 70% to class B (31,663 SNVs, 2,564 insertions and 1,798 deletions), suggesting that for a large part of these loci the reference allele could have phenotypic consequences.

**Table 2:** **GENCODE and ENCODE annotations of all the RRA loci for GRCh37 and GRCh38**

**Type**	**Total variants (GRCh37)**	**Class A**	**Class B**	**Class C**	**Total variants (GRCh38)**	**Class A**	**Class B**	**Class C**
Total SNVs	85111	17944	60714	6453	77274	11827	59055	6392
Intergenic	40321	8004	29053	3264	36774	5465	28072	3237
Gencode	44790	9940	31661	3189	40500	6362	30983	3155
Functional	580	167	373	40	402	31	335	36
Pseudogenes	734	135	518	81	633	68	487	78
Non coding RNA	8628	2040	5965	623	7673	1229	5828	616
Encode Elements	20438	4723	14138	1577	18458	3098	13811	1549
Total Insertions	6700	1870	4616	214	17	5	10	2
Intergenic	2912	751	2052	109	7	1	5	1
Gencode	3788	1119	2564	105	10	4	5	1
Functional	56	23	31	2	1	0	1	0
Pseudogenes	61	14	44	3	1	0	0	1
Non coding RNA	744	243	477	24	4	2	2	0
Encode Elements	1744	569	1122	53	4	1	3	0
Total Deletions	4462	1206	3095	161	3703	689	2857	157
Intergenic	1869	504	1298	67	1523	290	1168	65
Gencode	2593	702	1797	94	2180	399	1689	92
Functional	24	8	15	1	9	1	7	1
Pseudogenes	30	10	20	0	19	17	2	0
Non coding RNA	486	137	333	16	392	64	312	16
Encode Elements	1023	304	682	37	823	154	633	36

Columns report the total number of variants (Total Variants), the number of variants that belong to classes A, B, and C for all RRA SNVs and InDels in GRCh37 and GRCh38. Rows report the annotation features at which each RRA has been annotated.

To facilitate the interpretation of these RRA loci and discern between phenotypically relevant and neutral variants we used the Genomic Evolutionary Rate Profiling (GERP) “rejected substitutions” (RS) score (see Methods more details) and we calculated the total number of SNVs and insertions with RS score larger than 2 and the total number of deletions that contain at least one base with RS score larger than 2. Moreover, to study the functional consequence of RRA loci that belong to protein-coding genes, we annotated them as missense, nonsense, frameshift and splicing variants by using the Variant Effect Predictor (VEP) tool (see Methods). Among GENCODE RRAs, 4,104 SNVs and 745 InDels (408 insertions and 337 deletions) have RS ≥2 (Additional file 1: Table S1, number in brackets), and more than 90% belong to class A or B. Moreover, 580 SNVs and 80 InDels (56 insertions and 24 deletions) have functional consequences by changing or disrupting the protein sequence, and around 50% (289 SNVs and 44 InDels) of these belong to classes A or B and have or contain bases with RS ≥2. These results suggest that for more than 300 loci of the human reference genome in coding genes the reference allele could have loss-of-function or gain-of-function effects.

As a further step, to evaluate the impact of GENCODE RRA loci on rare and complex phenotypes, we exploited the annotation of the Genetic Association Database (GAD) and the Online Mendelian Inheritance in Man resource (OMIM) (see Methods). We found that around 25% of the genic RRA loci (8,704 SNVs, 707 insertions and 505 deletions) has positive association with a complex phenotype annotated at the GAD (Additional file 1: Figure S1.a-c) and 95% belong to class A or B. For both SNVs and InDels, the most represented GAD class is Psychiatric followed by Chemdependency, Neurological, Metabolic, Immune and Cardiovascular classes. Concerning ncRNAs (Additional file 1: Figure S1.d-f), 163 SNVs and 27 InDels (16 insertions and 11 deletions) are annotated at the GAD and the most represented categories are Cancer and Immune followed by Metabolic, Psychiatric, Developmental and Cardiovascular. Also for ncRNAs, around 95% of RRA loci belong to class A or B. Among the functionally relevant variants (missense, frameshift, nonsense and splicing), 113 (106 SNVs, 3 insertions and 4 deletions) belong to genes annotated in the OMIM database and 49 are of class A or B and have or contain bases with RS≥2.

#### Regulatory elements

Genomic elements involved in the regulation of transcription and in the processing and control of RNA transcripts play important cellular and developmental roles, and in some cases mutations within them contribute to diseases. Experimental evidences have shown that the presence of variants in regulatory regions can lead to differences in transcription factor (TF) binding between individuals [28], altering the regulation of gene transcription. This can be explained by the fact that variants can generate an increase or a decrease in the binding affinity of a given TF and leading to a change in gene expression. In some cases, SNVs and InDels may eliminate an existing binding site and/or generate a new one for a different TF, which can have a dramatic effect on the gene expression pattern.

For these reasons, to evaluate the impact of RRA loci on binding variation and regulatory mechanisms, we studied their overlap with the regulatory elements discovered by the ENCODE project (see Methods). We found that 20,438 SNVs and 2,767 small InDels (Additional file 1: Table S1) are part of at least one of the regulatory elements annotated by the ENCODE project. Furthermore, around 10% of these loci overlap with all the three different regulatory features (TFP, DHS and enhancers), 25% belong at least to two, while 65% are part of only one regulatory element. More than 90% of them (18,861 SNVs and 2,677 InDels) belong to class A or B (1,869 SNVs and 359 InDels with RS ≥2). GAD annotation of the total set of regulatory RRA loci showed that 2558 SNVs and 387 InDels are associated with a complex phenotype (Additional file 1: Figure S1.g-i). The reference allele of more than 90% of them has never been observed in homozygous state (class A or B). Also for regulatory RRA loci the most represented GAD phenotypes are Cardiovascular, Psychiatric, Metabolic and Neurological.

As a final step, we scored the regulatory impact of RRA loci by using the RegulomeDB tool [29]. RegulomeDB combines experimental datasets from ENCODE and other sources with computational predictions and manual annotations to score the functional impact of genomic variants. The scoring system represents with increasing confidence that a variant lies in a functional location and likely results in a functional consequence. Lower scores indicate increasing evidence for a variant to be located in a functional region (see Methods). These analyses are summarized in Additional file 1: Figure S1.j-l and show that 45,790 SNVs and 6731 InDels (3,959 insertions and 2,772 deletions) are predicted to have a functional role by RegulomeDB. In particular, 2,149 SNVs and 457 InDels (287 insertions and 170 deletions) are predicted to lie in a functional region and to affect TF binding (RegulomeDB score ≤3) and more than 90% belong to class A or B.

Taken as a whole, these results evidence that the GRCh37 assembly contains tens of thousands of loci located into coding, non coding and regulatory elements for which the presence of the reference allele may imply deleterious functional consequences. Missing these variants in re-sequencing studies could affect the interpretation of the whole results and hamper the discovery of disease-related loci.

### RRAs detection

**Figure 2:**
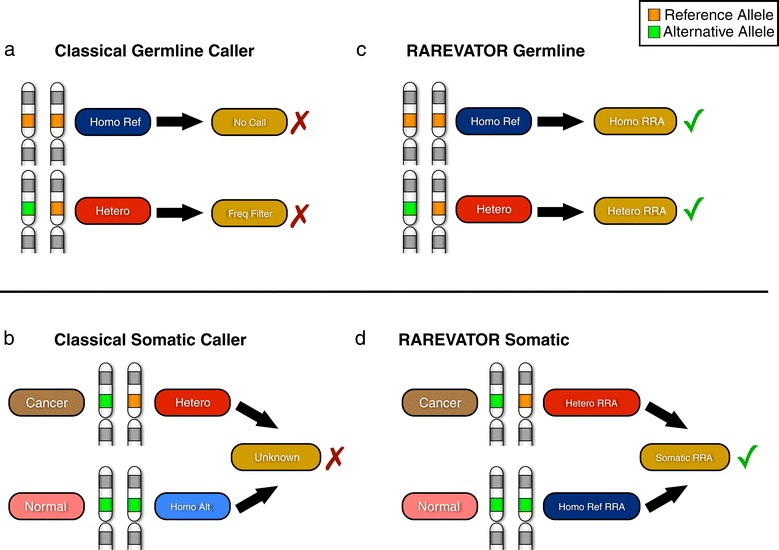
Variant calling scheme for classical SNP and InDel caller and RAREVATOR. Panels a and b report the calling scheme of classical germline and somatic variant callers. Panels c and d report the calling scheme of RAREVATOR for germline and somatic variants. Classical germline callers **(panel a)** are devised to detect variants from the comparison to the reference genome and, clearly, they are not able to detect variants in RRA loci when the reference allele is in homozygous state. Also heterozygous calls are missed by classical resequencing analysis pipelines, since variant calling is usually followed by a filtering step in which known variants with allele frequency larger than a predefined threshold (usually 0.01) are discarded. Somatic callers **(panel b)** are devised to detect every kind of differences between normal and tumor samples. However, the probabilistic calling step is followed by a filtering step in which germline variants found in normal sample are used to discard somatic calls. RAREVATOR exploits the GATK Unified Genotyper for interrogating all the RRA loci and detecting germline and somatic variants that contain the reference allele **(panels c and d)**.

The identification of SNVs and InDels from HTS data is accomplished by means of the so-called SNV and InDel callers that can be categorized into two main classes: germline and somatic methods. Somatic callers have been properly developed to detect variants occurring *de novo* within groups of somatic cells. To date, they have been mainly applied in cancer studies and the basic idea behind these methods consists in finding variant alleles that are present in the tumor sample but not in the matched control. The first computational method for somatic variants detection [16] relied on independently calling the genotype of the two samples followed by subtraction of the normal sample calls from the cancer calls to obtain a candidate somatic mutation set. Recently, several authors introduced somatic methods, such as VarScan2 [21] and Mutect [22], that use a probabilistic framework to simultaneously compare the reads of a tumor sample and a matched normal sample to identify statistically significant differences at each variant site. Since these methods are devised to detect every kind of differences between normal and tumor samples, in principle, they are able to identify somatic variants that belong to RRA loci. However, the probabilistic calling step is followed by a filtering step [21,22] in which germline variants found in normal sample are used to discard somatic calls (Figure 2). For this reason, in RRA loci, somatic variants in heterozygous state are rejected when the normal sample has the alternative allele (the most frequent allele) in homozygous state.

On the other hand, germline callers are devised to detect variants from the solely comparison to the reference genome. They are all based on probabilistic frameworks that use the number of reads presenting differences from the reference genome to detect a variant. Clearly, they are not able to detect variants in RRA loci when the reference allele is in homozygous state. Moreover, also the reference alleles in heterozygous state are missed by classical resequencing analysis pipelines, since variant calling is usually followed by a filtering step in which known variants [30,31] with allele frequency larger than a predefined threshold (usually 0.01) are discarded (Figure 2).

Following these considerations, currently available callers are not able to identify variants that contain the reference allele in RRA loci. To overcome these limits, we developed a novel tool, RAREVATOR (RAre REference Variants annotaTOR), that exploits the GATK Unified Genotyper for interrogating all the RRA loci and detecting, among them, variants that contain the reference allele (see Figure 2 and Methods). RAREVATOR takes into account only GATK high-quality calls (variant quality score, QUAL ≥ 30). The variant quality score is a Phred-scaled estimation of the calling confidence of GATK, and QUAL ≥ 30 corresponds to an error rate smaller than 0.1%. For this reason, the sensitivity and specificity of our tool completely reflect the performance of the algorithms at the base of the Unified Genotyper GATK computational pipeline. RAREVATOR is able to call germline RRA variants in single- and multi-samples experiments (germline mode) as well as somatic RRA variants in pairs of matched tumor and normal samples (somatic mode).

To demonstrate the usefulness of our tool in re-sequencing studies, we applied it to the analysis of a publicly available whole-exome sequencing dataset of 18 pairs of metastasizing uveal melanomas and matched peripheral blood lymphocytes samples [32]. We first applied RAREVATOR on the 18 peripheral blood samples for the identification of germline variants. This analysis (Additional file 1: Table S2) shows that classical germline calling pipelines missed the detection of 1476 RRA SNVs (778 in homozygous and 694 in heterozygous state) and 112 InDels (58 in homozygous and 54 in heterozygous state). More than 80% of these variants (1217 SNVs and 86 InDels) belong to class B, and only a small fraction are of class A (121 SNVs and 13 InDels) or class C (138 SNVs and 13 InDels). Among the RRAs calls we also found variants that lead to protein functional consequences (missense and splicing) and variants that have a positive association with a complex phenotype annotated at the GAD (25 SNVs are associated with CANCER phenotype).

Then we used RAREVATOR on the 18 matched tumor-normal pairs to identify somatic variants in RRA loci and we compared these results to those obtained by Mutect and VarScan2. Our tool was able to identify 22 somatic variants that were missed by the other two state-of-the-art methods, and 8 of these variants are missense (see Additional file 1: Table S2). RRA missense mutations affected five different genes: DCC, CR1, FAT2, GCC2 and CLCN1. To understand the relevance of these five genes in tumorigenesis we studied their role in other cancer studies by using the DriverDB database [33]. DriverDB is a web resource that includes mutation profiles from 6079 tumor-normal pairs (4397 from TCGA, 861 from ICGC, 112 from PCGP, 238 from TARGET and 471 from published papers) of 14 different cancer types and exploits eight computational methods to identify driver cancer genes for each tumor type. We downloaded all the driver genes inferred by the DriverDB resources and we found that all the five genes affected by missense RRAs in our uveal melanoma dataset were predicted as driver gene in at least one cancer type by one of the eight computational methods (Additional file 1: Table S3). Surprisingly, four of the genes containing RRA missense mutaions in Uveal Melanoma (DCC, CR1, GCC2 and CLCN1) were predicted as diver genes in TCGA skin cutaneous melanoma dataset. Although skin and uveal melanoma have different genetic and molecular features, are known to have the same cellular origin (they both derive from melanocytes) and the four genes predicted as driver in skin melanoma could be related to some key basic functions also in uveal melanoma.

To evaluate the potential involvement of the RRA somatic mutations on the uveal melanoma tumorigenesis, we searched for significantly mutated genes (SMG) and pathways (SMP) that contain the RRA mutations. SMGs are genes that have a higher mutation rate with respect to a background mutation rate [34]. A gene can be found to be a SMG because it contains driver mutations that are selected during tumorigenesis, and therefore can contribute to disease progression. In the same way, SMPs are pathways (or gene sets) with a high mutation rate and that are selected during tumorigenesis and can contribute to disease progression. To identify SMGs and SMPs, we applied the MutSigCV [35] and DrGaP [36] algorithms to the sets of somatic mutations identified by muTect and VarScan2 and separately to the same sets integrated with the RRA mutations identified by RAREVATOR. MutSigCV estimates the background mutation rate for each gene-patient-category combination based on the observed silent mutations in the gene and non-coding mutations in the surrounding regions and is considered one of the best methods for the identification of SMGs. DrGaP models the probability of observing a given set of mutations in a gene with the Poisson distribution and then calculates a Likelihood Ratio Test to evaluate if there is an increased rate of mutation. The Poisson model for a single gene is extended to analyze a pathway or gene set by treating multiple genes within a pathway as a “big” gene. We used DrGaP to test several curated pathways and computational gene sets (see Methods for more details). The MutSigCV did not identify any SMG in all the datasets we tested. However, the results of these analyses show that RRA variants affect calculation and significance of the mutation rate: Additional file 1: Table S4 shows that the five genes that contain missense RRA mutations have a significant increase in SMG ranking when the analysis comprised the mutations identified by RAREVATOR. The RRAs have the same effect also in the calculation of the significantly mutated pathways. DrGaP identified four SMPS that contain the RRA mutations (Table 3). All these pathways are related to the immune response and interestingly, one of them (the IMMUNE_SYSTEM pathway curated by the Reactome database) reached the statistical significant threshold (p ≤ 0.1) thanks to the contribution of RRA mutations. These results do not demonstrate at all that RRA mutations actively participate to the tumorigenesis in the Uveal Melanoma cancer, but the missing of these variants can seriously affect the identification of genes and pathways that might have an important role in tumorigenesis and cancer progression.

**Table 3:** **DrGaP pathways analysis**

**MSigDB category**	**Gene set name**	**Somatic variant caller**	**Gene**	**P-value**	**RAREVATOR P-value**
BP	SYSTEM PROCESS	VarScan	CLCN1	0.0054	0.0051
CM	MODULE 46	VarScan	CR1	0.0009	0.0009
CM	MODULE 75	VarScan	CR1	0.0032	0.0031
REACTOME	IMMUNE SYSTEM	muTect	CR1	0.0103	0.0099

Significantly mutated pathways identified by DrGaP algorithm by using the somatic mutations detected by MuTect and VarScan2 on the uveal melanoma dataset. The table reports the p-value calculated by DrGaP on MuTect and VarScan2 mutations, with and without the RRA variants. Columns report the MSigDB gene set category, the gene set name, the algorithm used for calling somatic variants, the RRA gene that belong to gene set, the DrGaP p-values calculated on the somatic variants (p-value) before and after the addition of the RRA variants detected by RAREVATOR (RAREVATOR P-value).

### RRAs in pathways and driver genes

The results described in previous section on a small cancer dataset demonstrated that the identification of mutations in RRA loci can affect the discovery of driver genes and pathways in cancer studies. To obtain a global picture of the potential impact of RRA loci on biological processes and functional pathways, we selected all the genes in the human genome that contains at least one functionally relevant RRA loci (602 genes with at least one splicing, missense, nonsense or frameshift variant) and we searched for enriched gene sets. The enrichment analysis was applied to the Gene Ontology (GO) terms Biological Process (BP), Molecular Function (MF), and Cellular Component (CC), and to pathway databases curated by KEGG, Reactome and Biocarta, by using the DAVID web resource [37,38]. The enrichment analysis identified 59 statistically significant gene sets (ease p-value ≤ 0.05): 21 GO CC terms, 9 MF terms, 21 BP terms, 4 Reactome and 3 KEGG pathways (Additional file 1: Table S5). Significantly enriched GO MF terms are mainly related to ion binding function, while many enriched BP terms are associated to lipid metabolism and transport. Interestingly, among BP terms and pathways, we observed a recurrent enrichment for functions related to cell adhesion and extracellular matrix (ECM) interaction, and for the DNA repair pathway. Strikingly, adhesion and interaction with extracellular matrix and surrounding cells are extremely important for integrity and homeostasis maintenance in normal tissues, and alterations in microenvironment and related functions are notoriously associated with cancer transformation and metastasis [39]. Moreover, the DNA repair pathway is one of the crucial processes responsible for genome integrity and cell survival. Strictly connected to cell cycle checkpoints, alterations of genes involved in this pathway support genetic instability and DNA damage tolerance, typically leading to death escape for tumor cells. Being this pathway a hallmark of cancer it is currently exploited for molecular targeted therapeutic approaches [40]. Overall, these results indicated that functionally relevant RRAs are involved in basic cellular processes essential for normal cell functions, whose impairment is strictly associated with malignant transformation and tumorigenesis.

**Figure 3:**
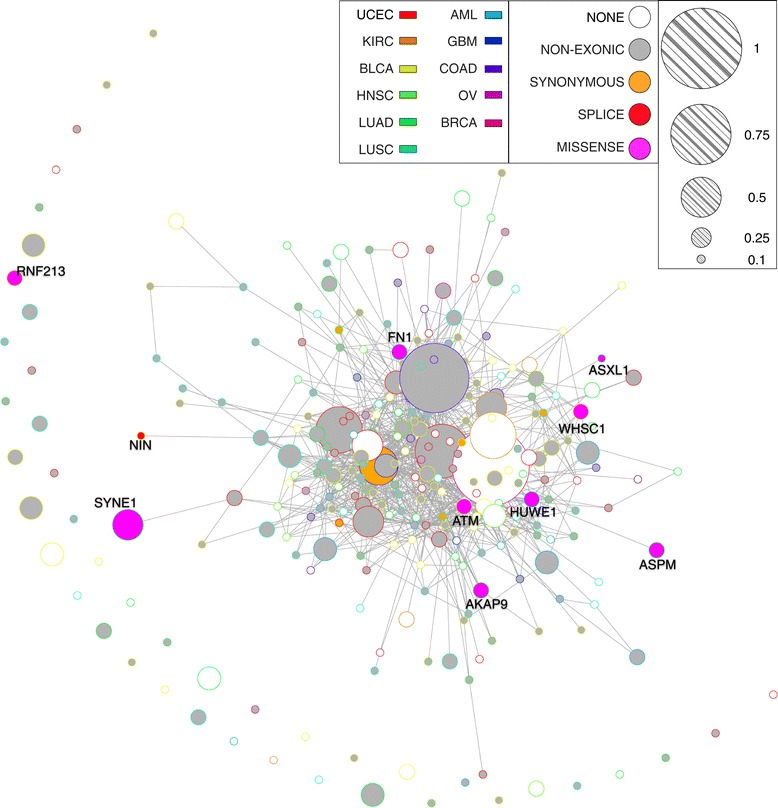
Network of TCGA driver genes containing RRA loci in GRCh37. To build the network we selected all the interactions of HumanNet v1 that link the 291 TCGA driver genes [41]. Node colors represent the most severe RRA variant contained by each gene: none RRA (white), non-exonic (grey), synonymous (orange), splicing (red) and missense RRA (magenta). Node border colors represent the TCGA cancer type for which the gene has been predicted to be a driver with maximum mutation frequency: AML (Acute Myeloid Leukemia), BLCA (Bladder Urothelial Carcinoma), BRCA (Breast invasive carcinoma), COAD (Colon adenocarcinoma), GBM (Glioblastoma multiforme), HNSC (Head and Neck squamous cell carcinoma), KIRC (Kidney renal clear cell carcinoma), LUAD (Lung adenocarcinoma), LUSC (Lung squamous cell carcinoma), OV (Ovarian serous cystadenocarcinoma), UCEC (Uterine Corpus Endometrial Carcinoma), UVM (Uveal Melanoma). Node radius gives a measure of the maximum mutation frequency of the driver gene in the TCGA cancer dataset coded by the border color.

To evaluate the potential impact of RRA loci in cancer studies, we assessed the overlap between genes containing RRA loci and the cancer driver genes predicted by TCGA. Recently, [41], by exploiting the TCGA whole-exome sequencing datasets (including 3,205 tumors from 12 different cancer types) and eight different algorithms for SMG identification, provided a list of 291 high-confidence cancer driver genes. Among them (Figure 3), we found that 187 genes (187/291, 64.2%) showed at least one RRA locus in exons, introns or UTR, while 10 genes (10/291, 3.4%) contained a functionally relevant RRA (nine missense and one splicing variant). All these genes with a missense or splicing RRA locus were predicted to be common driver genes in at least 11 or all the 12 cancer types taken into consideration, thus emphasizing their involvement in essential tumorigenic processes (Additional file 1: Table S6). Eight of these genes have a mutation frequency larger than 0.1 in at least one of the 12 cancer types. Notably, among them, we found the serine/threonine kinase PI3/PI4-kinase ATM gene, which encodes a crucial checkpoint kinase controlling several downstream proteins, including TP53 and BRCA1, required for cell cycle arrest and DNA damage repair activation. Given its centrality for many cancer types, this apical master controller is currently used as clinically actionable target for anti-cancer therapies [40]. Taken as a whole, these results demonstrated that RRA loci with potential functional consequences could impact genes and pathways that play fundamental roles in several disease states.

### GRCh38

Recently, the GRC announced the public release of GRCh38, the latest version of the human reference genome assembly. In the announcement (http://genomeref.blogspot.it), the GRC reports that “large scale studies of human variation, such as the 1000 Genomes Project, identified a number of bases and indels in GRCh37 that were never seen in any individuals, suggesting they may represent errors in the assembly.” For these reasons several thousand individual bases were updated in GRCh38.

To understand the extent of this update we compared the reference alleles between GRCh37 and GRCh38 assemblies and we found that the great majority of SNVs (77,274, 90.8%) and deletions (3,703, 83%) was not corrected, while almost all insertions (6,683, 99.7%) were updated (see Methods, Table 2 and Additional file 1: Table S7 for more details). Among the unchanged RRA loci we found 37,345 SNVs and 2,088 deletions of class A or B belonging to GENCODE features, and among them, 3,262 SNVs and 251 deletions have RS ≥2. Although a significant fraction of coding RRA loci of class A was corrected (see Table 2 and Additional file 1: Table S6), the GRCh38 assembly still contains 374 class A or B variants (366 SNVs and 8 deletions) with functional consequences and 68 (66 SNVs and 2 deletions) are located into OMIM genes.

Moreover, 10,917 SNVs and 545 deletions have a positive association with a GAD phenotype and as in the analysis of GRCh37, the most represented phenotypes are CHEMDEPENDENCY, CARDIOVASCULAR, METABOLIC and IMMUNE (Additional file 1: Figure S2.a-i). For regulatory elements (Additional file 1: Table S7), 18458 SNVs and 823 deletions are part of ENCODE features and of these variants, 90% (16,903 SNVs and 787 deletions) belongs to class A or B. Furthermore, 41,407 SNVs and 2,338 deletions are still annotated by RegulomeDB and around 5% have a score ≤3 (Additional file 1: Figure S2.j-l). Concerning cancer genes, 173 TCGA driver genes still contain at least one RRA locus, and as in GRCh37, for 10 of them the RRA is functionally relevant (Additional file 1: Figure S3 and Additional file 1: Table S6). Taken as a whole, these results show that although the GRC corrected almost all the RRA insertions and a significant fraction of SNVs and deletions for which the reference allele has never been observed, a large number of functionally relevant RRA loci still remain unchanged in GRCh38.

## Discussion and conclusion

The development of HTS technologies is revolutionizing the molecular diagnosis of human diseases. The ability to generate enormous amount of sequence data in a short time at an affordable cost makes this approach ideal for a wide range of applications from sequencing a group of candidate genes, all coding regions to the entire human genome. The identification of variants with these technologies is based on comparing the reads produced by HTS platforms with the haploid sequence of the human reference genome and finding the bases that differ from the reference genome sequence. HTS-based approaches have been successfully used to identify disease causing variants in mendelian disorders as well as in the study of genomic instability in cancer samples. In this work we demonstrate that the haploid sequence of the human reference genome GRCh37 contains almost 20000 loci in which the reference allele has never been observed in healthy individuals and around 70000 loci in which it has been observed only in the heterozygous state. We studied the genomic complexity of the regions surrounding these loci and the geographical origins of their alleles and we found that their presence in the human reference assembly can be due to both sequencing errors and rare/private variants of the anonymous individuals used by the Human Genome Project. By analyzing the genomic position of these loci we found that more than half of them belong to GENCODE biotypes and around 25% are part of regulatory elements discovered by the ENCODE project.

Moreover, we also discovered that 1% of these loci belongs to coding portions of the genome and that a significant fraction has potential functional consequences on genes that have been previously linked to Mendelian disorders, complex diseases and cancer. The complete list of these functionally relevant RRA loci is reported in Additional files 2 and 3. In these tables the genomic information is enriched with functional and disease annotations (from RegulomeDB, GAD and OMIM databases). Among the OMIM annotation of the genes containing functionally relevant SNV and InDel RRA loci, we found genetic phenotypes causing developmental delay, intellectual disability/mental retardation, cardiomyopathy and cancer (prostate, ovarian, breast and leukemia).

To emphasize the impact that many of these loci could have on human health, we report some examples of RRA variants present in genes that have been demonstrated to be causative of neurological disorders and cancer. The ASPM gene, that is associated with primary autosomal recessive microcephaly type 5 and seizures [42,43], contains a missense RRA SNV of class B. Another missense RRA SNV of class B belongs to FAT4 gene, whose biallelic disruption was recently associated with a human recessive syndrome including periventricular neuronal heterotopia [44]. GRIA3, that is an important gene associated with a X-linked intellectual disability known as lissencephaly, and RECQL4, that is responsible for some cases of the autosomal recessive Rothmund-Thomson syndrome, contain a frameshift insertion and a splicing insertion of class B, respectively. Among class A variants, we found missense, splicing and frameshift RRA loci in COL4A1, PIGN and ATM genes. COL4A1 has been associated to several disorders and has been reported to carry causal heterozygous mutations in patients with porencephaly and in patients with schizencephaly [45]. PIGN encodes a protein involved in glycosylphosphatidylinositol-anchor biosynthesis, and recently a missense mutation has been found in patients with an autosomal recessive syndrome characterized by dysmorphic features and multiple congenital anomalies together with severe neurological impairment, chorea and seizures leading to early death [46]. ATM gene encodes a protein belonging to the PI3/PI4-kinase family and functions as a regulator of a wide variety of downstream proteins, including tumor suppressor proteins p53 and BRCA1. Somatic mutations in this gene have been identified in T-cell prolymphocytic leukemia, mantle cell lymphoma, and B-cell chronic lymphocytic leukemia [47], while germline mutations have been found associated to increased susceptibility to early-onset breast cancer [48] and risk of glioma and meningioma [49].

This brief list of variants which contains genes associated with severe disorders might represent an underestimate of the relevance of RRA. The analyses reported in the results section show that there is huge amount of other genes and regulatory regions containing RRA. Failure to identify these RRA could seriously limit the identification of disease-causing variants in Mendelian and polygenic disorders and driver genes in cancer studies.

To overcome the limits of currently available variant callers, we developed therefore RAREVATOR, a novel tool that interrogates all the RRA loci and detects variants that contain the reference allele. RAREVATOR is able to identify germline variants in single- and multi-samples experiments (germline mode) as well as somatic variants in pairs of matched tumor and normal samples (somatic mode). The use of our tool on a publicly available whole-exome sequencing cancer dataset demonstrated its uniqueness in the identification of germline and somatic variants: RAREVATOR was able to identify more than 1500 germline and 22 somatic RRA variants that we demonstrated to participate to significantly mutated pathways. The results obtained by RAREVATOR on this very small dataset emphasize the importance of our tool for reanalyzing the huge amount of HTS data generated in the last few years and aligned against the human reference genome GRCh37 for complementing the results obtained with classical variant callers in HTS-based studies. Moreover, although the GRC corrected almost all the RRA insertions and a significant fraction of SNVs and deletions for which the reference allele has never been observed, a large number of functionally relevant RRAs still remain unchanged in the latest version of the human reference genome. This will make of RAREVATOR a valuable instrument for complementing the results of classical variant caller also for the analysis of future sequencing data that will be aligned against the GRCh38 assembly.

## Methods

### Genome project data analysis

The 1000GP [4] is the first project aiming to sequence genomes of a large number of people, to provide a comprehensive resource on human genetic variation and find most genetic variants that have frequencies of at least 1% in the populations studied. The 1000GP consortium, by combining low-coverage whole-genome sequencing and high-coverage whole-exome sequencing of 1092 individuals from 14 populations drawn from Europe, East Asia, sub-Saharan Africa and the Americas, has identified around 38 million single nucleotide polymorphic positions, 1.4 million short insertions and deletions and more than 14,000 larger deletions. All the variant calls are stored in VCF format and freely available at the 1000GP FTP site. We downloaded Variant calls from ftp://ftp-trace.ncbi.nih.gov/1000genomes/ftp/release/20110521/, we filtered out structural variants and we calculated allele frequency for all SNVs and InDels by using the VCFTools [50] --freq option. We found that 85111 SNVs and 11162 InDels loci have a reference allele frequency smaller than 0.01. As a further step, in order to study the genotype frequency of these loci across the 1092 individuals, we converted the genotype data by using the --012 of the VCFTools. We found that 6453 SNVs and 375 InDels have all the three genotypes represented across the 1092 individuals, 17,944 SNVs and 3,076 InDels are present only in homozygous alternative state and 60,714 SNVs and 7,711 InDels appear in heterozygous and homozygous-alternative state.

### GC-content and mappability

For each RRA variant we selected a 100 bp region surrounding the locus and we calculated GC content and mappability. GC content was calculated by using the ’nuc’ option of the bedtools package [51], while the mappability was calculated by using the UCSC ’bigWigAverageOverBed’ program and the mappability track generated by the GEM mapper aligner [52]. This track provides a measure of the uniqueness of a sequence within the human genome. By using the GEM mapper aligner, where up to two mismatches were allowed, the method is equivalent to mapping sliding windows of k-mers back to the genome (where k has been set to 36, 40, 50, 75 or 100 nucleotides to produce these tracks). For each window, a mappability score was computed as S = 1/(number of matches found in the genome). S =1 means one match in the genome, S =0.5 is two matches in the genome, and so on). For our analysis we used the wgEncodeCrgMapabilityAlign100mer.bigWig (with k equal to 100 nucleotides) file that we downloaded at ftp://hgdownload.cse.ucsc.edu/gbdb/hg19/bbi/.

### Exone Sequencing Project (ESP)

ESP is a NHLBI project that aims to discover novel genes and mechanisms contributing to heart, lung and blood disorders by pioneering the application of next-generation sequencing of the protein-coding regions of the human genome across diverse, richly-phenotyped populations and to share these datasets and findings with the scientific community to extend and enrich the diagnosis, management and treatment of related disorders. The current ESP data release (ESP6500SI-V2) is obtained from 6503 samples drawn from multiple ESP cohorts and represents all of the ESP exome variant data. Sequences were aligned to NCBI build 37 human genome reference using BWA. PCR duplicates were removed using Picard. Alignments were recalibrated using GATK. Lane-level indel realignments and base alignment quality (BAQ) adjustments were applied. Allele frequency data for the ESP6500SI-V2 release were downloaded from http://evs.gs.washington.edu/evs_bulk_data/ESP6500SI-V2-SSA137.protein-hgvs-update.snps_indels.vcf.tar.gz.

### GENCODE

The GENCODE Consortium [19] aims to identify all gene features in the human genome using a combination of computational analysis, manual annotation, and experimental validation. Current version of the GENCODE (version 19) contains the annotation of 57,820 genes (20,345 Protein-coding genes, 13,870 Long non-coding RNA genes, 9,013 Small non-coding RNA genes and 14,206 Pseudogenes) and has more than 30,000 coding transcripts not represented in UCSC genes and RefSeq databases. It also has the most comprehensive annotation for long non-coding RNA (lncRNA) loci publicly available with the predominant transcript form consisting of two exons. GENCODE 19 is publicly available from www.gencodegenes.org.

### Functional annotation with VEP

For each RRA loci, functional annotation was performed by using the Variant Effect Predictor (VEP) tool. The VEP was previously known as the Ensembl SNP Effect Predictor [53]. The VEP tool can be used to quickly and accurately predict the effect of variants (SNPs, insertions, deletions, CNVs or structural variants) on genes, transcripts, and protein sequences, as well as regulatory regions. It is available as a Web interface (http://www.ensembl.org/Homo_sapiens/Tools/VEP) and a stand-alone perl script (https://github.com/Ensembl/ensembl-tools/archive/release/75.zip). All RRA loci were annotated by using the web interface of VEP.

### GERP

Genomic Evolutionary Rate Profiling (GERP) is a method for producing position-specific estimates of evolutionary constraint using maximum likelihood evolutionary rate estimation [54]. Given a multiple sequence alignment and a phylogenetic tree with branch lengths representing the neutral rate between the species within that alignment, GERP quantifies constraint intensity at each individual position in terms of rejected substitutions, the difference between the neutral rate and the estimated evolutionary rate at the position. Constraint intensity at each individual alignment position is quantified in terms of a “rejected substitutions” (RS) score, defined as the number of substitutions expected under neutrality minus the number of substitutions “observed” at the position. Positive scores represent a substitution deficit and thus indicate that a site may be under evolutionary constraint, while negative scores indicate that a site is probably evolving neutrally. GERP RS scores in BigWig format for the human reference genome hg19 were downloaded at http://hgdownload.cse.ucsc.edu/gbdb/hg19/bbi/All_hg19_RS.bw. The RS scores for each position in the human genome were generated by using GERP++ [55] to analyze the Threaded Blockset Aligner [56] alignment of hg19 to 35 other mammalian species (the most distant mammalian species being platypus), spanning over 3 billion positions. The alignment was compressed to remove gaps in the human sequence, and GERP++ scores were computed for every position with at least 3 ungapped species present. Importantly, the human sequence was removed from the alignment and not included in either the neutral rate estimation or the site-specific “observed” estimates, and therefore it is not included in the RS score. This is done to eliminate the confounding influence of deleterious derived alleles segregating in the human population that are present in the reference sequence and allowed to evaluate at our best the evolutionary constraint of our set of RRAs. In order to discern between phenotypically relevant and neutral variants we calculated the total number of SNVs and insertions with RS score greater than 2 and the total number of deletions that contain at least one base with RS score larger than 2. We chose 2 as RS cutoff because in [55] Davydov and colleagues found that coding exons exhibit the strongest levels of evolutionary constraint with an average RS score of around 2.

### Genetic Association Database (GAD)

The Genetic Association Database (GAD) [57] is an archive of human genetic association studies of complex diseases and disorders. This includes summary data extracted from published papers in peer reviewed journals on candidate gene and GWAS studies. The goal of this database is to allow the user to rapidly identify medically relevant polymorphisms from the large volume of polymorphism and mutational data, in the context of standardized nomenclature. GAD currently contains approximately 40,000 individual gene records of genetic association studies taken from over 23,000 independent publications. Importantly, a large number (11,568) of the records in GAD has a designation of whether the gene of record was reported to be associated (Y) or not (N) associated with the disease phenotype for that specific record. Many records, for various reasons, do not have such a designation. Genomic variants are associated to phenotypic descriptions captured at multiple levels. The top level is made of 19 “disease class” that comprise aging, cancer, cardiovascular, chemdependency, developmental, hematological, immune, infection, metabolic, mitochondrial, neurological, normalvariation, pharmacogenomic, psych, renal, reproduction, vision other or unknown (if the author did not identify a disease class). GAD annotation was downloaded in txt format from http://geneticassociationdb.nih.gov/.

### ENCODE

The main goal of the Encyclopedia of DNA Elements (ENCODE) project is to identify all functional elements in the human genome, including coding and non-coding transcripts, marks of accessible chromatin, and protein-binding sites (The ENCODE Project Consortium 2004, 2007, 2011). The data sets generated by the ENCODE Consortium are therefore particularly well suited for the functional interpretation of genomic variants identified in resequencing studies. To date, a total of 147 different cell types have been studied using a wide variety of experimental assays [20]. Chromatin accessibility has been studied using DNase-seq, which led to the identification of 2.89 million DNase Iypersensitive sites that may exhibit regulatory function. DNase footprinting was used to detect binding between proteins and the genome at a nucleotide resolution. To study the overlap between RRAs and regulatory elements, we used the annotation data produced by [58]. TF peaks, motifs, DHSs and enhancers are the same as used in ENCODE Integrative paper [20]. In total, there are 88 sequence-specific TFs (TFSSs), 16 general TFs (like Pol2- and Pol3- associated factors), and 15 chromatin-associated factors. A union set of enhancer elements is used consisting of those obtained using ChromHMM/Segway segmentation and distal regulatory modules obtained by discriminative training. All the annotation files were downloaded from: http://funseq.gersteinlab.org/data/.

### RegulomeDB

RegulomeDB [29] is a very powerful tool that guides the interpretation of regulatory variants in the human genome by combining experimental datasets from ENCODE and other sources with computational predictions and manual annotations.

RegulomeDB makes use of large sets of data including the following:

- Manually curated regions that have been experimentally characterized to be involved in regulation
- ChIP-seq information for a variety of important regulatory factors across a diverse set of cell types
- chromatin state information across over 100 cell types
- expression quantitative trait loci (eQTL) information allowing the association of distal sites with gene promoters

RegulomeDB currently includes all available ENCODE transcription factor (TF) ChIP-seq, histone ChIP-seq, FAIRE, and DNase I hypersensitive site data, transcription factor ChIP-seq data available from the NCBI Sequence Read Archive, a large collection of eQTL, dsQTL and ChIP-exo data. A total of 962 experimental datasets are included, covering over 100 tissues and cells lines and representing nearly 60 million annotations. These high-throughput data sources are supplemented through manual curation of literature sources. These provide valuable information from low-throughput but high-quality assays to aid in assigning function to SNVs. As an initial release, RegulomeDB contained manual curation from 97 papers focused on six loci, resulting in 188 genomic annotations. It also included 1448 validated enhancer regions from the VISTA Enhancer Browser and 855 SNVs shown to directly affect NFKB and RNA Pol 2 binding in lymphoblastoid cells. RegulomeDB authors developed a heuristic scoring system based on functional confidence of a variant. The scoring system represents with increasing confidence that a variant lies in a functional location and likely results in a functional consequence. Lower scores indicate increasing evidence for a variant to be located in a functional region. We applied RegulomeDB to all RRA loci for GRCh37 and GRCh38 assemblies and we summarized the results in the barplots of Additional file 1: Figure S1.j-l and Figure S2.j-l.

### RAREVATOR

RAREVATOR is a Perl script that executes the UnifiedGenotyper module of GATK for genotyping all the SNVs and InDels that belong to RRA loci and a series of R scripts that have been devised to filter and annotate the resulting variants. The UnifiedGenotyper module of GATK is a multiple-sample, technology-aware SNV and InDel caller. It uses a Bayesian genotype likelihood model to estimate simultaneously the most likely genotypes and allele frequency in a population of N samples, emitting an accurate posterior probability of there being a segregating variant allele at each locus as well as for the genotype of each sample. When the --output_mode argument of GATK is set to “EMIT_ALL_SITES”, the UnifiedGenotyper produces calls at any site specified in a.BED file. Moreover, when --genotyping_mode is set to “GENOTYPE_GIVEN_ALLELES” the genotype calls are forced to use only the alleles provided in a VCF file. Thanks to these output settings, RAREVATOR is able to make genotype calls for all the RRA loci by using the exact position and the alleles of the 85111 SNVs and the 11162 InDels stored in BED and VCF files. Once the results of the genotype calls have been stored in two VCF files (a file for SNVs and a file for InDels), R scripts are used to make germline or somatic calls for RRA positions in which the reference allele is present. When RAREVATOR is set in germline mode (--germline), the R scripts analyze one sample at a time by removing all the GATK low quality calls, all the variants that do not contain the reference allele and stores the results in a VCF file. On the other hand, when the tool is set on somatic mode (--somatic), the R script compares the calls made by GATK for each pair of matched tumor and normal samples. For each RRA locus, if the reference allele is not present in the normal sample and is present in the tumor sample, then a Fisher exact test is applied on the normal and tumor reads aligned at that locus and if the p-value is statistically significant (<0.01) then the RRA somatic variant is annotated and stored in a tab-delimited file. A tab-delimited file for each pair of normal-tumor samples is generated. Each variant called by RAREVATOR is annotated by using the VEP annotation combined with GENCODE, GAD and RegulomeDB annotation. RAREVATOR is freely available at http://sourceforge.net/projects/rarevator. The package includes the Perl and R codes, the VCF and BED files with all the RRA variants informations and the manual describing how to use the tool in detail.

### DriverDB, Significantly mutated genes and pathways

DriverDB (http://ngs.ym.edu.tw/driverdb/) is a database which incorporates 6079 cases of exome-seq data, annotation databases (such as dbSNP, 1000 Genome and Cosmic) and bioinformatics algorithms dedicated to driver gene/mutation identification. DriverDB provide two points of view, “Cancer” and “Gene”. The “Cancer” section summarizes the calculated results of driver genes by eight computational methods for a specific cancer type/dataset and provides three levels of biological interpretation for realization of the relationships between driver genes. The “Gene” section is designed to visualize the mutation information of a driver gene in five different aspects. We interrogated all the tumor types of the Cancer section of the database and we downloaded the lists of driver genes identified by each of the eight driver identification tools.

MutSigCV [35] analyzes lists of mutations discovered in DNA sequencing, to identify genes that are mutated more often than expected by chance given background mutation processes. The input data to MutSigCV is lists of mutations (and indels) from a set of samples (patients) that were subjected to DNA sequencing, as well as information about how much of the genomic region is covered in the sequencing. To create the input lists of mutations for MutSig, we annotated the muTect and VarScan2 somatic variants with the VEP annotator and we then converted the VCF annotated files in mac format by using the vcf2maf tool (https://github.com/ckandoth/vcf2maf). We applied MutSigCV with default settings on somatic mutations identified by VarScan2 and muTect with and without the somatic RRA variants detected by RAREVATOR. DrGaP [36] is a powerful and flexible statistical framework for identifying driver genes and driver signaling pathways in cancer genome sequencing studies. We downloaded DrGaP at http://code.google.com/p/drgap/ and we applied it with default settings to the lists of somatic mutations identified by muTect and VarScan2 and on the same lists augmented with the RAREVATOR somatic calls. We used DrGaP to test pathways and computational gene sets curated by Molecular Signatures Database (MSigDB, http://www.broadinstitute.org/gsea/msigdb/). The MSigDB is a collection of annotated gene sets that are divided into 7 major collections, and several subcollections. For DrGaP analysis we used the three Gene Ontology sets (CC, BP, MF), the pathways curated by Biocarta, KEGG and REACTOME and the 431 cancer modules.

### TCGA drivers interaction network

The interaction network of Figure 3 was built by using the HumanNet v1 [59] as backbone. In [59], Lee et al. developed a Bayesian statistical method that allows for the evaluation of functional associations between gene products by integrating many heterogeneous functional data. The HumanNet v1 is a probabilistic functional gene network made of 18714 validated protein-coding genes of Homo sapiens, constructed by Bayesian integration of 21 types of ’omics’ data from multiple organisms, with each data type weighted according to how well it links genes that are known to function together in Homo sapiens. To build the networks of Figure 3 and Additional file 1: Figure S3 we selected all the interactions of HumanNet v1 that link the 291 TCGA driver genes. Colors nodes are assigned according to the class of RRA variant they contain and color border according to the cancer type for which the gene was predicted as driver.

### GRCh38

GRCh38 represents the first major assembly update since GRCh37 in 2009, and introduces changes to chromosome coordinates. We downloaded the GRCh38 assembly from the GenBank FTP site: ftp://ftp.ncbi.nlm.nih.gov/genbank/genomes/Eukaryotes/vertebrates_mammals/Homo_sapiens/GRCh38/. To understand the extent of this update with respect to the set of loci studied in this work, we remapped the genomic positions of all the RRA loci from GRCh37 to the GRCh38 assembly by using the NCBI Genome Remapping Service (http://www.ncbi.nlm.nih.gov/genome/tools/remap).

## Additional files

Additional file 1
**Supplemental methods.** Supplemental methods to Characterization and identification of hidden rare variants in the human genome.

Additional file 2
**Functionally relevant RRA SNVs.** Tab-delimited Table of missense, nonsense and splicing RRA SNVs.

Additional file 3
**Functionally relevant RRA InDels.** Tab-delimited Table of Frameshift and splicing RRA InDels.

## References

[1] Wheeler DA, Srinivasan M, Egholm M, Shen Y, Chen L, McGuire A (2008). The complete genome of an individual by massively parallel dna sequencing. Nature..

[2] Bentley DR, Balasubramanian S, Swerdlow HP, Smith GP, Milton J, Brown CG (2008). Accurate whole human genome sequencing using reversible terminator chemistry. Nature..

[3] McKernan KJ, Peckham HE, Costa GL, McLaughlin SF, Fu Y, Tsung EF (2009). Sequence and structural variation in a human genome uncovered by short-read, massively parallel ligation sequencing using two-base encoding. Genome Res..

[4] Abecasis GR, Altshuler D, Auton A, Brooks LD, Durbin RM, 1000 Genomes Project Consortium (2010). A map of human genome variation from population-scale sequencing. Nature..

[5] Chin L, Andersen JN, Futreal PA (2011). Cancer genomics: from discovery science to personalized medicine. Nat Med..

[6] Topol EJ (2013). From dissecting cadavers to dissecting genomes. Sci Transl Med..

[7] Snyder M, Du J, Gerstein M (2010). Personal genome sequencing: current approaches and challenges. Genes Dev..

[8] Li H, Durbin R (2009). Fast and accurate short read alignment with burrows-wheeler transform. Bioinformatics..

[9] Langmead B, Trapnell C, Pop M, Salzberg SL (2009). Ultrafast and memory-efficient alignment of short dna sequences to the human genome. Genome Biol.

[10] Li H, Handsaker B, Wysoker A, Fennell T, Ruan J, Homer N (2009). 1000 Genome Project Data Processing Subgroup. The sequence alignment/map format and samtools. Bioinformatics..

[11] McKenna A, Hanna M, Banks E, Sivachenko A, Cibulskis K, Kernytsky A (2010). The genome analysis toolkit: a mapreduce framework for analyzing next-generation dna sequencing data. Genome Res..

[12] Albers CA, Lunter G, MacArthur DG, McVean G, Ouwehand WH, Durbin R (2011). Dindel: accurate indel calls from short-read data. Genome Res..

[13] Yoon S, Xuan Z, Makarov V, Ye K, Sebat J (2009). Sensitive and accurate detection of copy number variants using read depth of coverage. Genome Res..

[14] Magi A, Tattini L, Cifola I, D’Aurizio R, Benelli M, Mangano E (2013). Excavator: detecting copy number variants from whole-exome sequencing data. Genome Biol..

[15] Ng SB, Buckingham KJ, Lee C, Bigham AW, Tabor HK, Dent KM (2010). Exome sequencing identifies the cause of a mendelian disorder. Nat Genet..

[16] Pleasance ED, Cheetham RK, Stephens PJ, McBride DJ, Humphray SJ, Greenman CD (2010). A comprehensive catalogue of somatic mutations from a human cancer genome. Nature..

[17] International Human Genome Sequencing Consortium (2004). Finishing the euchromatic sequence of the human genome. Nature..

[18] Abecasis GR, Auton A, Brooks LD, DePristo MA, Durbin RM, 1000 Genomes Project Consortium (2012). An integrated map of genetic variation from 1,092 human genomes. Nature..

[19] Harrow J, Frankish A, Gonzalez JM, Tapanari E, Diekhans M, Kokocinski F (2012). Gencode: the reference human genome annotation for the encode project. Genome Res..

[20] ENCODE Project Consortium (2012). An integrated encyclopedia of dna elements in the human genome. Nature.

[21] Koboldt DC, Zhang Q, Larson DE, Shen D, McLellan MD, Lin L (2012). Varscan 2: somatic mutation and copy number alteration discovery in cancer by exome sequencing. Genome Res..

[22] Cibulskis K, Lawrence MS, Carter SL, Sivachenko A, Jaffe D, Sougnez C (2013). Sensitive detection of somatic point mutations in impure and heterogeneous cancer samples. Nat Biotechnol..

[23] Osoegawa K, Mammoser AG, Wu C, Frengen E, Zeng C, Catanese JJ (2001). A bacterial artificial chromosome library for sequencing the complete human genome. Genome Res..

[24] Deanna Church on the Reference Genome Past, Present and Future. http://www.bio-itworld.com/2013/4/22/church-on-reference-genomes-past-present-future.html.

[25] Ball EV, Stenson PD, Abeysinghe SS, Krawczak M, Cooper DN, Chuzhanova NA (2005). Microdeletions and microinsertions causing human genetic disease: common mechanisms of mutagenesis and the role of local dna sequence complexity. Hum Mutat..

[26] Stenson PD, Ball EV, Mort M, Phillips AD, Shiel JA, Thomas NST (2003). Human gene mutation database (hgmd): 2003 update. Hum Mutat..

[27] Esteller M (2011). Non-coding rnas in human disease. Nat Rev Genet..

[28] Kasowski M, Grubert F, Heffelfinger C, Hariharan M, Asabere A, Waszak SM (2010). Variation in transcription factor binding among humans. Science..

[29] Boyle AP, Hong EL, Hariharan M, Cheng Y, Schaub MA, Kasowski M (2012). Annotation of functional variation in personal genomes using regulomedb. Genome Res..

[30] Gilissen C, Hoischen A, Brunner HG, Veltman JA (2012). Disease gene identification strategies for exome sequencing. Eur J Hum Genet..

[31] Kanchi KL, Johnson KJ, Lu C, McLellan MD, Leiserson MDM, Wendl MC (2014). Integrated analysis of germline and somatic variants in ovarian cancer. Nat Commun..

[32] Harbour JW, Onken MD, Roberson EDO, Duan S, Cao L, Worley LA (2010). Frequent mutation of bap1 in metastasizing uveal melanomas. Science..

[33] Cheng W-C, Chung I-F, Chen C-Y, Sun H-J, Fen J-J, Tang W-C (2014). Driverdb: an exome sequencing database for cancer driver gene identification. Nucleic Acids Res..

[34] Youn A, Simon R (2011). Identifying cancer driver genes in tumor genome sequencing studies. Bioinformatics..

[35] Lawrence MS, Stojanov P, Polak P, Kryukov GV, Cibulskis K, Sivachenko A (2013). Mutational heterogeneity in cancer and the search for new cancer-associated genes. Nature..

[36] Hua X, Xu H, Yang Y, Zhu J, Liu P, Lu Y (2013). Drgap: a powerful tool for identifying driver genes and pathways in cancer sequencing studies. Am J Hum Genet.

[37] Huang DW, Sherman BT, Lempicki RA (2009). Systematic and integrative analysis of large gene lists using david bioinformatics resources. Nat Protoc..

[38] Huang DW, Sherman BT, Lempicki RA (2009). Bioinformatics enrichment tools: paths toward the comprehensive functional analysis of large gene lists. Nucleic Acids Res..

[39] Cox TR, Erler JT (2011). Remodeling and homeostasis of the extracellular matrix: implications for fibrotic diseases and cancer. Dis Model Mech..

[40] Weber AM, Ryan AJ. Atm and atr as therapeutic targets in cancer. Pharmacol Ther. 2014. doi:10.1016/j.pharmthera.2014.12.001. 10.1016/j.pharmthera.2014.12.001 25512053

[41] Tamborero D, Gonzalez-Perez A, Perez-Llamas C, Deu-Pons J, Kandoth C, Reimand J (2013). Comprehensive identification of mutational cancer driver genes across 12 tumor types. Sci Rep..

[42] Bond J, Roberts E, Mochida GH, Hampshire DJ, Scott S, Askham JM (2002). Aspm is a major determinant of cerebral cortical size. Nature Genet..

[43] Shen J, Eyaid W, Mochida GH, Al-Moayyad F, Bodell A, Woods CG (2005). Aspm mutations identified in patients with primary microcephaly and seizures. J Med Genet..

[44] Cappello S, Gray MJ, Badouel C, Lange S, Einsiedler M, Srour M (2013). Mutations in genes encoding the cadherin receptor-ligand pair dchs1 and fat4 disrupt cerebral cortical development. Nat Genet..

[45] Yoneda Y, Haginoya K, Kato M, Osaka H, Yokochi K, Arai H (2013). Phenotypic spectrum of col4a1 mutations: porencephaly to schizencephaly. Ann Neurol..

[46] Maydan G, Noyman I, Har-Zahav A, Neriah ZB, Pasmanik-Chor M, Yeheskel A (2011). Multiple congenital anomalies-hypotonia-seizures syndrome is caused by a mutation in pign. J Med Genet..

[47] Grønbaek K, Worm J, Ralfkiaer E, Ahrenkiel V, Hokland P, Guldberg P (2002). Atm mutations are associated with inactivation of the arf-tp53 tumor suppressor pathway in diffuse large b-cell lymphoma. Blood..

[48] Brunet J, Gutiérrez-Enríquez S, Torres A, Bérez V, Sanjosé S, Galceran J (2008). Atm germline mutations in spanish early-onset breast cancer patients negative for brca1/brca2 mutations. Clin Genet..

[49] Malmer BS, Feychting M, Lönn S, Lindström S, Grönberg H, Ahlbom A (2007). Genetic variation in p53 and atm haplotypes and risk of glioma and meningioma. J Neurooncol..

[50] Danecek P, Auton A, Abecasis G, Albers CA, Banks E, DePristo MA (2011). 1000 Genomes Project Analysis Group. The variant call format and vcftools. Bioinformatics..

[51] Quinlan AR, Hall IM (2010). Bedtools: a flexible suite of utilities for comparing genomic features. Bioinformatics..

[52] Marco-Sola S, Sammeth M, Guigó R, Ribeca P (2012). The gem mapper: fast, accurate and versatile alignment by filtration. Nat Methods..

[53] McLaren W, Pritchard B, Rios D, Chen Y, Flicek P, Cunningham F (2010). Deriving the consequences of genomic variants with the ensembl api and snp effect predictor. Bioinformatics..

[54] Cooper GM, Stone EA, Asimenos G, Green ED, Batzoglou S, NISC Comparative Sequencing Program (2005). Distribution and intensity of constraint in mammalian genomic sequence. Genome Res..

[55] Davydov EV, Goode DL, Sirota M, Cooper GM, Sidow A, Batzoglou S (2010). Identifying a high fraction of the human genome to be under selective constraint using gerp++. PLoS Comput Biol..

[56] Blanchette M, Kent WJ, Riemer C, Elnitski L, Smit AFA, Roskin KM (2004). Aligning multiple genomic sequences with the threaded blockset aligner. Genome Res..

[57] Becker KG, Barnes KC, Bright TJ, Wang SA (2004). The genetic association database. Nat Genet..

[58] Khurana E, Fu Y, Colonna V, Mu XJ, Kang HM, Lappalainen T (2013). Integrative annotation of variants from 1092 humans: application to cancer genomics. Science..

[59] Lee I, Blom UM, Wang PI, Shim JE, Marcotte EM (2011). Prioritizing candidate disease genes by network-based boosting of genome-wide association data. Genome Res..

