# Supplementary material for: Characterization and identification of hidden rare variants in the human genome
Source: BMC Genomics. 2015 Apr 24;16(1):340. doi: 10.1186/s12864-015-1481-9 (PMC4416239; doi:10.1186/s12864-015-1481-9)
Supplement: Additional file 1 — Supplemental methods. Supplemental methods to Characterization and identification of hidden rare variants in the human genome. [file 12864_2015_1481_MOESM1_ESM.pdf]

Supplemental Methods to "Characterization and identification of hidden rare variants in the human genome".

Alberto Magi[1,*], Romina D'Aurizio[2], Flavia Palombo[3], Ingrid Cifola[4], Lorenzo Tattini[5], Roberto Semeraro[1], Tommaso Pippucci[3], Betti Giusti[1], Giovanni Romeo[3], Rosanna Abbate[1] and Gian Franco Gensini[1].

[1]Department of Clinical and Experimental Medicine, University of Florence, Florence, Italy.
[2]Institute of Informatics and Telematics and Institute of Clinical Physiology, National Research Council, Pisa, Italy.
[3]Medical Genetics Unit, Department of Medical and Surgical Sciences, University of Bologna, Bologna, Italy.
[4]Institute for Biomedical Technologies, National Research Council, Segrate, Milano, Italy.
[5]Department of Neuroscience, Pharmacology and Child Health, University of Florence, Florence, Italy.

Email: Alberto Magi*- albertomagi@gmail.com;

*Corresponding author

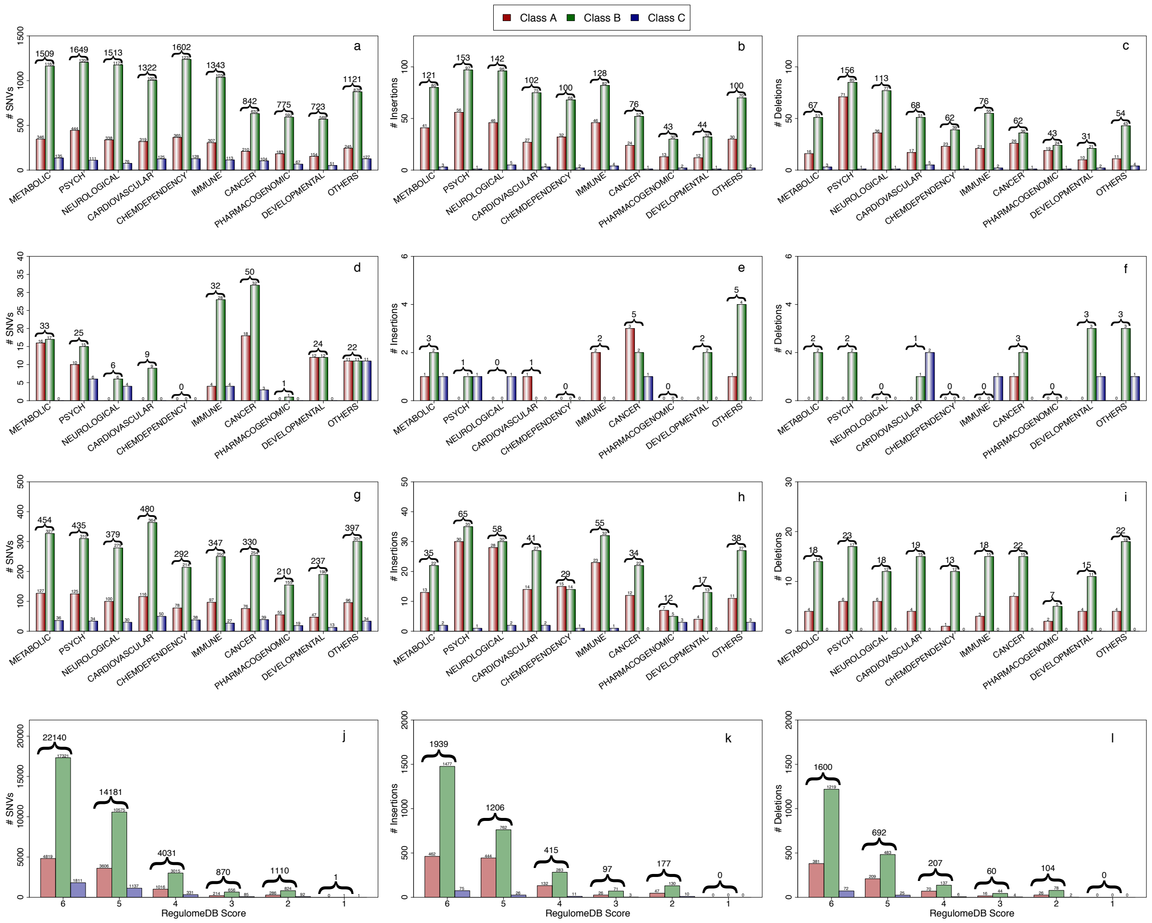

Supplemental Figure 1: GAD and RegulomeDB annotation of GRCh37 RRAs loci. Panels a, b and c report the GAD annotation of SNVs (a), Insertions (b) and Deletions (c) that belong to Protein-coding genes. Panels d, e and f report the GAD annotation of SNVs, Insertions and Deletions that belong to ncRNA biotypes. Each variant can be annotated to multiple GAD phenotype. Panels g, h and i report the GAD annotation of SNVs (g), Insertions (h) and Deletions (i) that belong to regulatory elements annotated at ENCODE. Panels j, k and l report the total number of SNVs (j), Insertions (k) and Deletions (l) as a function of RegulomeDB score. In each panel of the figure, RRA loci have been classified as belonging to class A, B and C (see main text for more details). Numbers on the top of curly brackets represent the sum of classes A and B variants.

Table 1: GENCODE and ENCODE annotations of all the RRAs loci for GRCh37.

Type	Total Variants	Class A	Class B	Classes A+B	Class C
Total SNVs	85111 (6478)	17944 (1555)	60714 (4456)	78658 (6011)	6453 (467)
Intergenic	40321 (2374)	8004 (502)	29053 (1692)	37057 (2194)	3264 (180)
Gencode	44790 (4104)	9940 (1053)	31661 (2764)	41601 (3817)	3189 (287)
Genic*	35428 (3441)	7765 (870)	25178 (2335)	32943 (3205)	2485 (236)
Intronic	33521 (2853)	7310 (693)	23862 (1965)	31172 (2658)	2349 (195)
UTR	1103 (226)	253 (57)	770 (151)	1023 (208)	80 (18)
Coding	804 (362)	202 (120)	546 (219)	748 (339)	56 (23)
Functional	580 (306)	167 (110)	373 (179)	540 (289)	40 (17)
missense	476 (272)	138 (97)	305 (160)	443 (257)	33 (15)
splicing	98 (30)	27 (11)	66 (18)	92 (28)	6 (2)
nonsense	6 (4)	2 (2)	3 (2)	5 (4)	1 (0)
OMIM	106 (53)	28 (16)	72 (33)	100 (49)	6 (4)
Pseudogenes	734 (38)	135 (7)	518 (26)	653 (33)	81 (5)
non coding RNA	8628 (625)	2040 (176)	5965 (403)	8005 (579)	623 (46)
lincRNA	5363 (419)	1282 (120)	3669 (264)	4951 (384)	412 (35)
antisense	1698 (108)	395 (26)	1212 (76)	1607 (102)	91 (6)
PT	1228 (79)	288 (28)	842 (47)	1130 (75)	98 (4)
NMD	194 (12)	49 (2)	132 (9)	181 (11)	13 (1)
SO	120 (6)	24 (0)	90 (6)	114 (6)	6 (0)
Other ncRNA	25 (1)	2 (0)	20 (1)	22 (1)	3 (0)
Encode Elements	20438 (2010)	4723 (495)	14138 (1374)	18861 (1869)	1577 (141)
TFP	5199 (464)	1209 (114)	3596 (319)	4805 (433)	394 (31)
DHS	7660 (669)	1768 (157)	5261 (453)	7029 (610)	631 (59)
Enhancer	992 (89)	221 (19)	698 (65)	919 (84)	73 (5)
TFP-DHS	2459 (300)	536 (69)	1734 (213)	2270 (282)	189 (18)
TFP-Enhancer	1878 (200)	446 (57)	1300 (135)	1746 (192)	132 (8)
DHS-Enhancer	516 (44)	135 (13)	348 (30)	483 (43)	33 (1)
TFP-DHS-Enhancer	1734 (244)	408 (66)	1201 (159)	1609 (225)	125 (19)
Total Insertions	6700 (619)	1870 (236)	4616 (370)	6486 (606)	214 (13)
Intergenic	2912 (211)	751 (71)	2052 (131)	2803 (202)	109 (9)
Gencode	3788 (408)	1119 (165)	2564 (239)	3683 (404)	105 (4)
Genic*	2983 (330)	862 (130)	2043 (199)	2905 (329)	78 (1)
Intronic	2819 (272)	807 (107)	1937 (164)	2744 (271)	75 (1)
UTR	119 (34)	33 (9)	84 (25)	117 (34)	2 (0)
Coding	45 (24)	22 (14)	22 (10)	44 (24)	1 (0)
Functional	56 (28)	23 (13)	31 (14)	54 (27)	2 (1)
splicing	14 (5)	4 (0)	9 (4)	13 (4)	1 (1)
Frameshift	42 (23)	19 (13)	22 (10)	41 (23)	1 (0)
OMIM	3 (0)	1 (0)	2 (0)	3 (0)	0 (0)
Pseudogenes	61 (4)	14 (0)	44 (3)	58 (3)	3 (1)
non coding RNA	744 (74)	243 (35)	477 (37)	720 (72)	24 (2)
lincRNA	404 (31)	119 (14)	268 (17)	387 (31)	17 (0)
antisense	146 (14)	56 (7)	89 (7)	145 (14)	1 (0)
PT	126 (20)	36 (7)	84 (11)	120 (18)	6 (2)
NMD	48 (8)	28 (7)	20 (1)	48 (8)	0 (0)
SO	17 (1)	4 (0)	13 (1)	17 (1)	0 (0)
Other ncRNA	3 (0)	0 (0)	3 (0)	3 (0)	0 (0)
Encode Elements	1744 (208)	569 (69)	1122 (134)	1691 (203)	53 (5)
TFP	509 (64)	159 (24)	342 (40)	501 (64)	8 (0)
DHS	484 (50)	173 (18)	291 (31)	464 (49)	20 (1)
Enhancer	83 (7)	26 (2)	55 (4)	81 (6)	2 (1)
TFP-DHS	200 (22)	75 (9)	120 (12)	195 (21)	5 (1)
TFP-Enhancer	236 (32)	73 (10)	157 (20)	230 (30)	6 (2)
DHS-Enhancer	39 (1)	14 (0)	23 (1)	37 (1)	2 (0)
TFP-DHS-Enhancer	193 (32)	49 (6)	134 (26)	183 (32)	10 (0)
Total Deletions	4462 (494)	1206 (145)	3095 (327)	4301 (472)	161 (22)
Intergenic	1869 (157)	504 (44)	1298 (103)	1802 (147)	67 (10)
Gencode	2593 (337)	702 (101)	1797 (224)	2499 (325)	94 (12)
Genic*	2077 (290)	555 (84)	1444 (195)	1999 (279)	78 (11)
Intronic	2006 (257)	536 (76)	1395 (172)	1931 (248)	75 (9)
UTR	51 (19)	12 (1)	36 (16)	48 (17)	3 (2)
Coding	20 (14)	7 (7)	13 (7)	20 (14)	0 (0)
Functional	24 (15)	8 (7)	15 (7)	23 (14)	1 (1)
splicing	6 (3)	2 (1)	3 (1)	5 (2)	1 (1)
Frameshift	18 (12)	6 (6)	12 (6)	18 (12)	0 (0)
OMIM	4 (2)	0 (0)	4 (2)	4 (2)	0 (0)
Pseudogenes	30 (4)	10 (3)	20 (1)	30 (4)	0 (0)
non coding RNA	486 (43)	137 (14)	333 (28)	470 (42)	16 (1)
lincRNA	278 (22)	87 (8)	181 (13)	268 (21)	10 (1)
antisense	115 (12)	26 (4)	85 (8)	111 (12)	4 (0)
PT	74 (7)	20 (2)	52 (5)	72 (7)	2 (0)
NMD	11 (1)	4 (0)	7 (1)	11 (1)	0 (0)
SO	5 (1)	0 (0)	5 (1)	5 (1)	0 (0)
Other ncRNA	3 (0)	0 (0)	3 (0)	3 (0)	0 (0)
Encode Elements	1023 (162)	304 (44)	682 (112)	986 (156)	37 (6)
TFP	322 (53)	95 (15)	220 (35)	315 (50)	7 (3)
DHS	302 (40)	96 (10)	195 (29)	291 (39)	11 (1)
Enhancer	63 (9)	20 (3)	38 (5)	58 (8)	5 (1)
TFP-DHS	114 (23)	29 (8)	81 (15)	110 (23)	4 (0)
TFP-Enhancer	126 (21)	36 (5)	83 (15)	119 (20)	7 (1)
DHS-Enhancer	24 (2)	9 (0)	14 (2)	23 (2)	1 (0)
TFP-DHS-Enhancer	72 (14)	19 (3)	51 (11)	70 (14)	2 (0)

Columns report the total number of variants (Total Variants), the number of variants that belong to classes A, B, A+B and C for all RRA SNVs and InDels. Rows report the GENCODE and ENCODE features at which each RRA has been annotated. For each variant type, in parenthesis are reported the total number of RRAs with RS score larger than 2.

Table 2: Germline and Somatic variants identified by RAREVATOR, MuTect and VarScan2 in the Uveal Melanomas dataset.

Category	Homozygoous	Heterozygous	Somatic	VarScan	Mutect
Total SNVs	778	698	22	0	0
Class A	52	69	1	0	0
UTR	0	2	0	0	0
Synonymous	0	1	0	0	0
Missense	0	2	0	0	0
Splicing	0	0	0	0	0
Nonsense	0	0	0	0	0
Class B	665	552	18	0	0
UTR	15	15	0	0	0
Synonymous	1	17	4	0	0
Missense	1	27	6	0	0
Splicing	0	7	0	0	0
Nonsense	0	0	0	0	0
Class C	61	77	3	0	0
UTR	0	2	1	0	0
Synonymous	0	4	0	0	0
Missense	0	3	2	0	0
Splicing	0	0	0	0	0
Nonsense	0	0	0	0	0
GAD	75	97	4	0	0
GADCancer	9	16	0	0	0
ENCODE	265	300	14	0	0
Total InDels	58	54	0	0	-
Class A	5	8	0	0	-
UTR	0	0	0	0	-
Non-Frameshift	0	0	0	0	-
Splicing	0	0	0	0	-
Frameshift	0	0	0	0	-
Class B	47	39	0	0	-
UTR	0	6	0	0	-
Non-Frameshift	0	0	0	0	-
Splicing	0	0	0	0	-
Frameshift	0	0	0	0	-
Class C	6	7	0	0	-
UTR	0	1	0	0	-
Non-Frameshift	0	0	0	0	-
Splicing	2	0	0	0	-
Frameshift	0	0	0	0	-
GAD	8	8	0	0	-
GADCancer	0	0	0	0	-
ENCODE	11	18	0	0	-

Columns report the total number of variants (Total Variants), the number of variants that belong to classes A, B, A+B and C for all RRA SNVs and InDels. Rows report the GENCODE and ENCODE features at which each RRA has been annotated.

Table 3: DriverDB prediction. Genes with missense RRA somatic mutations in Uveal Melanoma and predicted as driver by the DriverDB resource.

Cancer Type	DCC	CR1	GCC2	FAT2	CLCN1
Acute Myeloid Leukemia	-	-	-	-	-
Bladder Urothlial Carcinoma	-	-	Dendrix	-	-
Breast invasive carcinoma	NetBox	NetBox	-	-	-
Colon adenocarcinoma	Dendrix, MDPFinder, NetBox	-	-	-	
Glioblastoma multiforme	NetBox	-	-	-	-
Kidney renal papillary cell carcinoma	-	-	-	-	
Lung adenocarcinoma	NetBox	-	-	-	Simon
Lung squamous cell carcinoma	NetBox	-	-	-	-
Ovarian serous cystadenocarcinoma	NetBox	-	-	-	-
Rectum adenocarcinoma	Dendrix, NetBox	-	-	Dendrix	-
Skin cutaneous melanoma	NetBox, Simon	NetBox	OncodriveFM	-	Simon
Stomach adenocarcinoma	NetBox	NetBox	-	-	-
Thyroid carcinoma	-	-	-	-	-
Uterine Corpus Endometrioid Carcinoma	NetBox	-	-	-	-

Columns report the cancer type and the RRA genes of Uveal Melanoma. For each gene and cancer type we report the algorithms that predicted that gene as a driver gene.

Table 4: MutSig analysis results on Uveal Melanoma dataset. The table reports the p-values calculated by the MutSig algorithm for the five genes with RRA missense mutations. The analyses have been performed by using the somatic mutations detected by MuTect and VarScan2, with and without the RRA variants.

Somatic Caller	DCC	CR1	GCC2	FAT2	CLCN1
muTect	0.188 (1620)	0.633 (4753)	0.659 (4902)	0.344 (2840)	0.313 (2595)
muTect + RAREVATOR	0.099 (907)	0.39 (3194)	0.545 (4266)	0.168 (1461)	0.169 (1476)
VarScan	0.982 (4393)	0.998 (5010)	0.989 (4548)	1 (5383)	0.98 (4355)
VarScan + RAREVATOR	0.949 (3979)	0.99 (4568)	0.983 (4405)	1 (5382)	0.945 (3951)

Columns report the genes with missense somatic RRA mutations in Uveal Melanoma, while rows reports the somatic caller used for generating the list of mutations for the MutSigCV analysis. In brackets is reported the ranking of each gene in the MutSig results.

Table 5: GO terms and pathways significantly enriched of genes that contains functionally relevant RRA loci.

Database	ID	Name	Gene Number	p-value	Gene Number (GRCh38)	p-value (GRCh38)
REACTOME	REACT_13552	Integrin cell surface interactions	8	0.0077	7	0.0133
REACTOME	REACT_216	DNA Repair	8	0.0252	7	0.0369
REACTOME	REACT_604	Hemostasis	13	0.0334	13	0.0107
REACTOME	REACT_18266	Axon guidance	5	0.0497	5	0.0305
REACTOME	REACT_15314	Hormone biosynthesis	5	0.0595	5	0.0369
KEGG	hsa04512	ECM-receptor interaction	9	0.0016	8	0.0025
KEGG	hsa04510	Focal adhesion	14	0.0024	13	0.0015
KEGG	hsa00230	Purine metabolism	9	0.0497	8	0.053
KEGG	hsa00980	Metabolism of xenobiotics by cytochrome P450	5	0.0741	5	0.0435
KEGG	hsa00150	Androgen and estrogen metabolism	4	0.0739	4	0.0481
GOTERM_BP	GO:0022610	biological adhesion	34	0.0028	24	0.0578
GOTERM_BP	GO:0007155	cell adhesion	34	0.0028	24	0.059
GOTERM_BP	GO:0051180	vitamin transport	5	0.0042	5	0.0021
GOTERM_BP	GO:0042157	lipoprotein metabolic process	8	0.0073	7	0.0105
GOTERM_BP	GO:0051642	centrosome localization	3	0.0075	3	0.0051
GOTERM_BP	GO:0006869	lipid transport	11	0.0081	8	0.0516
GOTERM_BP	GO:0046942	carboxylic acid transport	11	0.0089	10	0.0072
GOTERM_BP	GO:0015849	organic acid transport	11	0.0093	10	0.0075
GOTERM_BP	GO:0042159	lipoprotein catabolic process	3	0.0111	3	0.0075
GOTERM_BP	GO:0016337	cell-cell adhesion	16	0.0119	14	0.012
GOTERM_BP	GO:0010876	lipid localization	11	0.0137	8	0.0725
GOTERM_BP	GO:0031348	negative regulation of defense response	5	0.0181	-	-
GOTERM_BP	GO:0008202	steroid metabolic process	12	0.0285	10	0.0455
GOTERM_BP	GO:0015721	bile acid and bile salt transport	3	0.0308	3	0.0212
GOTERM_BP	GO:0042953	lipoprotein transport	3	0.0308	-	-
GOTERM_BP	GO:0007156	homophilic cell adhesion	9	0.0323	8	0.0326
GOTERM_BP	GO:0001894	tissue homeostasis	6	0.0323	6	0.0151
GOTERM_BP	GO:0010743	regulation of foam cell differentiation	4	0.0324	-	-
GOTERM_BP	GO:0007160	cell-matrix adhesion	7	0.04	-	-
GOTERM_BP	GO:0008203	cholesterol metabolic process	7	0.0458	-	-
GOTERM_BP	GO:0007229	integrin-mediated signaling pathway	6	0.0476	5	0.0788
GOTERM_BP	GO:0030384	phosphoinositide metabolic process	6	0.0552	6	0.0269
GOTERM_BP	GO:0006928	cell motion	20	0.0804	19	0.0273
GOTERM_BP	GO:0048854	brain morphogenesis	3	0.0505	3	0.0352
GOTERM_BP	GO:0060249	anatomical structure homeostasis	7	0.0795	7	0.0364
GOTERM_BP	GO:0042158	lipoprotein biosynthetic process	5	0.0809	5	0.045
GOTERM_BP	GO:0048871	multicellular organismal homeostasis	6	0.0924	6	0.0471
GOTERM_MF	GO:0005509	calcium ion binding	48	0.0001	41	0.0003
GOTERM_MF	GO:0051183	vitamin transporter activity	4	0.0045	4	0.0028
GOTERM_MF	GO:0046872	metal ion binding	145	0.0076	124	0.0079
GOTERM_MF	GO:0043167	ion binding	148	0.0078	127	0.007
GOTERM_MF	GO:0043169	cation binding	146	0.0081	125	0.008
GOTERM_MF	GO:0008092	cytoskeletal protein binding	25	0.0137	22	0.0138
GOTERM_MF	GO:0015293	symporter activity	10	0.0196	10	0.007
GOTERM_MF	GO:0004000	adenosine deaminase activity	3	0.0214	-	-
GOTERM_MF	GO:0005201	extracellular matrix structural constituent	7	0.0406	6	0.0618
GOTERM_MF	GO:0003779	actin binding	16	0.0576	16	0.0158
GOTERM_MF	GO:0001883	purine nucleoside binding	57	0.0914	52	0.0335
GOTERM_MF	GO:0001882	nucleoside binding	-	NA	52	0.0373
GOTERM_MF	GO:0030554	adenyl nucleotide binding	56	0.0972	51	0.0378
GOTERM_MF	GO:0016887	ATPase activity	16	0.068	15	0.0384
GOTERM_MF	GO:0015370	solute:sodium symporter activity	5	0.0657	5	0.0392
GOTERM_CC	GO:0044430	cytoskeletal part	46	0.0006	39	0.0012
GOTERM_CC	GO:0005578	proteinaceous extracellular matrix	21	0.0009	16	0.0101
GOTERM_CC	GO:0031012	extracellular matrix	22	0.001	16	0.0186
GOTERM_CC	GO:0044421	extracellular region part	44	0.0023	34	0.0214
GOTERM_CC	GO:0030016	myofibril	10	0.0046	9	0.0051
GOTERM_CC	GO:0015629	actin cytoskeleton	17	0.0048	16	0.002
GOTERM_CC	GO:0044449	contractile fiber part	10	0.0052	9	0.0056
GOTERM_CC	GO:0044420	extracellular matrix part	10	0.0065	9	0.0069
GOTERM_CC	GO:0043292	contractile fiber	10	0.0081	9	0.0084
GOTERM_CC	GO:0030018	Z disc	6	0.0099	6	0.0046
GOTERM_CC	GO:0015630	microtubule cytoskeleton	26	0.0155	23	0.0123
GOTERM_CC	GO:0031674	I band	6	0.0204	6	0.0098
GOTERM_CC	GO:0030017	sarcomere	8	0.0226	8	0.0089
GOTERM_CC	GO:0005604	basement membrane	7	0.0244	7	0.0107
GOTERM_CC	GO:0005856	cytoskeleton	53	0.0247	45	0.028
GOTERM_CC	GO:0030139	endocytic vesicle	6	0.0268	5	0.052
GOTERM_CC	GO:0044463	cell projection part	13	0.0378	11	0.0543
GOTERM_CC	GO:0016324	apical plasma membrane	9	0.0379	9	0.0144
GOTERM_CC	GO:0005615	extracellular space	29	0.0382	24	0.0626
GOTERM_CC	GO:0005886	plasma membrane	125	0.0415	102	0.0947
GOTERM_CC	GO:0044433	cytoplasmic vesicle part	11	0.0435	9	0.0794
GOTERM_CC	GO:0009897	external side of plasma membrane	10	0.0566	10	0.0208
GOTERM_CC	GO:0005874	microtubule	14	0.053	13	0.032
GOTERM_CC	GO:0042995	cell projection	28	0.0696	25	0.0453

Columns report the annotation database (Database), the code of the significant pathway/term (ID), the name of the pathway/term (Name), the number of RRA genes that belong to pathway/term (Gene Number) and the enrichment p-value calculated by the DAVID (p-value).

Table 6: TCGA driver genes with a functionally relevant RRA locus. The table reports the list of genes predicted as driver by Tambonero et al. that contain a missense or splicing RRA locus in GRCh37 and GRCh38.

Driver Gene	Variant Type	N Cancer	Cancer Types	Mutation Frequency
ASPM	missense	11	LUNG ADENO	0.175
FN1	missense	12	LUNG SQUAMOUS CELL	0.109
WHSC1	missense	11	BLADDER UROTHELIA	0.10
SYNE1	missense	12	LUNG SQUAMOUS CELL	0.34
AKAP9	missense	11	LUNG ADENO	0.10
ATM	missense	11	BLADDER UROTHELIAL	0.12
RNF213	missense	12	BLADDER UROTHELIAL	0.10
ASXL1	missense	12	LUNG SQUAMOUS CELL	0.06
HUWE1	missense	11	LUNG SQUAMOUS CELL	0.12
NIN	splicing	12	BLADDER UROTHELIAL	0.08

Columns report the TCGA driver genes that contain the RRA locus (Driver Gene), the variant type of the RRA locus, the total number of TCGA cancer type in which the gene has been predicted as driver, the cancer type with maximum mutation frequency and the mutation frequency.

Table 7: GENCODE and ENCODE annotations of all the RRAs loci for GRCh38.

Type	Total Variants	Class A	Class B	Classes A+B	Class C
Total SNVs	77274 (5736)	11827 (951)	59055 (4325)	70882 (5276)	6392 (460)
Intergenic	36774 (2194)	5465 (358)	28072 (1656)	33537 (2014)	3237 (180)
Gencode	40500 (3542)	6362 (593)	30983 (2669)	37345 (3262)	3155 (280)
Genic*	32194 (2973)	5065 (488)	24668 (2255)	29733 (2743)	2461 (230)
Intronic	30663 (2537)	4893 (428)	23436 (1916)	28329 (2344)	2334 (193)
UTR	960 (196)	136 (32)	744 (146)	880 (178)	80 (18)
Coding	571 (240)	36 (28)	488 (193)	524 (221)	47 (19)
Functional	402 (194)	31 (24)	335 (156)	366 (180)	36 (14)
missense	331 (176)	27 (22)	275 (142)	302 (164)	29 (12)
splicing	69 (18)	4 (2)	59 (14)	63 (16)	6 (2)
nonsense	2 (0)	0 (0)	1 (0)	1 (0)	1 (0)
OMIM	70 (35)	3 (3)	63 (30)	66 (33)	4 (2)
Pseudogenes	633 (33)	68 (4)	487 (24)	555 (28)	78 (5)
non coding RNA	7673 (536)	1229 (101)	5828 (390)	7057 (491)	616 (45)
lincRNA	4770 (359)	769 (70)	3592 (254)	4361 (324)	409 (35)
antisense	1513 (92)	241 (14)	1185 (73)	1426 (87)	87 (5)
PT	1094 (66)	178 (15)	818 (47)	996 (62)	98 (4)
NMD	169 (12)	29 (2)	127 (9)	156 (11)	13 (1)
SO	104 (6)	11 (0)	87 (6)	98 (6)	6 (0)
Other ncRNA	23 (1)	1 (0)	19 (1)	20 (1)	3 (0)
Encode Elements	18458 (1784)	3098 (304)	13811 (1340)	16909 (1644)	1549 (140)
TFP	4709 (412)	820 (69)	3506 (312)	4326 (381)	383 (31)
DHS	6895 (602)	1146 (104)	5128 (440)	6274 (544)	621 (58)
Enhancer	909 (75)	146 (6)	690 (64)	836 (70)	73 (5)
TFP-DHS	2231 (279)	352 (50)	1696 (211)	2048 (261)	183 (18)
TFP-Enhancer	1702 (170)	294 (31)	1276 (131)	1570 (162)	132 (8)
DHS-Enhancer	467 (38)	89 (7)	345 (30)	434 (37)	33 (1)
TFP-DHS-Enhancer	1545 (208)	251 (37)	1170 (152)	1421 (189)	124 (19)
Total Insertions	17 (2)	5 (0)	10 (1)	15 (1)	2 (1)
Intergenic	7 (0)	1 (0)	5 (0)	6 (0)	1 (0)
Gencode	10 (2)	4 (0)	5 (1)	9 (1)	1 (1)
Genic*	5 (0)	2 (0)	3 (0)	5 (0)	0 (0)
Intronic	4 (0)	1 (0)	3 (0)	4 (0)	0 (0)
UTR	1 (0)	1 (0)	0 (0)	1 (0)	0 (0)
Coding	0 (0)	0 (0)	0 (0)	0 (0)	0 (0)
Functional	1 (0)	0 (0)	1 (0)	1 (0)	0 (0)
splicing	1 (0)	0 (0)	1 (0)	1 (0)	0 (0)
Frameshift	0 (0)	0 (0)	0 (0)	0 (0)	0 (0)
OMIM	1 (0)	0 (0)	1 (0)	1 (0)	0 (0)
Pseudogenes	1 (1)	0 (0)	0 (0)	0 (0)	1 (1)
non coding RNA	4 (1)	2 (0)	2 (1)	4 (1)	0 (0)
lincRNA	0 (0)	0 (0)	0 (0)	0 (0)	0 (0)
antisense	0 (0)	0 (0)	0 (0)	0 (0)	0 (0)
PT	4 (1)	2 (0)	2 (1)	4 (1)	0 (0)
NMD	0 (0)	0 (0)	0 (0)	0 (0)	0 (0)
SO	0 (0)	0 (0)	0 (0)	0 (0)	0 (0)
Other ncRNA	0 (0)	0 (0)	0 (0)	0 (0)	0 (0)
Encode Elements	4 (0)	1 (0)	3 (0)	4 (0)	0 (0)
TFP	1 (0)	1 (0)	0 (0)	1 (0)	0 (0)
DHS	0 (0)	0 (0)	0 (0)	0 (0)	0 (0)
Enhancer	0 (0)	0 (0)	0 (0)	0 (0)	0 (0)
TFP-DHS	0 (0)	0 (0)	0 (0)	0 (0)	0 (0)
TFP-Enhancer	0 (0)	0 (0)	0 (0)	0 (0)	0 (0)
DHS-Enhancer	0 (0)	0 (0)	0 (0)	0 (0)	0 (0)
TFP-DHS-Enhancer	3 (0)	0 (0)	3 (0)	3 (0)	0 (0)
Total Deletions	3703 (399)	689 (78)	2857 (299)	3546 (377)	157 (22)
Intergenic	1523 (136)	290 (29)	1168 (97)	1458 (126)	65 (10)
Gencode	2180 (263)	399 (49)	1689 (202)	2088 (251)	92 (12)
Genic*	1769 (236)	318 (45)	1375 (180)	1693 (225)	76 (11)
Intronic	1725 (218)	313 (44)	1339 (165)	1652 (209)	73 (9)
UTR	38 (15)	4 (0)	31 (13)	35 (13)	3 (2)
Coding	6 (3)	1 (1)	5 (2)	6 (3)	0 (0)
Functional	9 (4)	1 (1)	7 (2)	8 (3)	1 (1)
splicing	4 (2)	0 (0)	3 (1)	3 (1)	1 (1)
Frameshift	5 (2)	1 (1)	4 (1)	5 (2)	0 (0)
OMIM	2 (0)	0 (0)	2 (0)	2 (0)	0 (0)
Pseudogenes	19 (0)	17 (0)	2 (0)	19 (0)	0 (0)
non coding RNA	392 (27)	64 (4)	312 (22)	376 (26)	16 (1)
lincRNA	224 (13)	42 (2)	172 (10)	214 (12)	10 (1)
antisense	99 (9)	12 (1)	83 (8)	95 (9)	4 (0)
PT	51 (3)	7 (1)	42 (2)	49 (3)	2 (0)
NMD	10 (1)	3 (0)	7 (1)	10 (1)	0 (0)
SO	5 (1)	0 (0)	5 (1)	5 (1)	0 (0)
Other ncRNA	3 (0)	0 (0)	3 (0)	3 (0)	0 (0)
Encode Elements	823 (127)	154 (19)	633 (102)	787 (121)	36 (6)
TFP	256 (44)	52 (9)	197 (32)	249 (41)	7 (3)
DHS	236 (33)	43 (4)	183 (28)	226 (32)	10 (1)
Enhancer	53 (5)	11 (0)	37 (4)	48 (4)	5 (1)
TFP-DHS	94 (17)	16 (4)	74 (13)	90 (17)	4 (0)
TFP-Enhancer	107 (17)	19 (2)	81 (14)	100 (16)	7 (1)
DHS-Enhancer	19 (2)	4 (0)	14 (2)	18 (2)	1 (0)
TFP-DHS-Enhancer	58 (9)	9 (0)	47 (9)	56 (9)	2 (0)

Columns report the total number of variants (Total Variants), the number of variants that belong to classes A, B, A+B and C for all RRA SNVs and InDels. Rows report the GENCODE and ENCODE features at which each RRA has been annotated. For each variant type, in parenthesis are reported the total number of RRAs with RS score larger than 2.

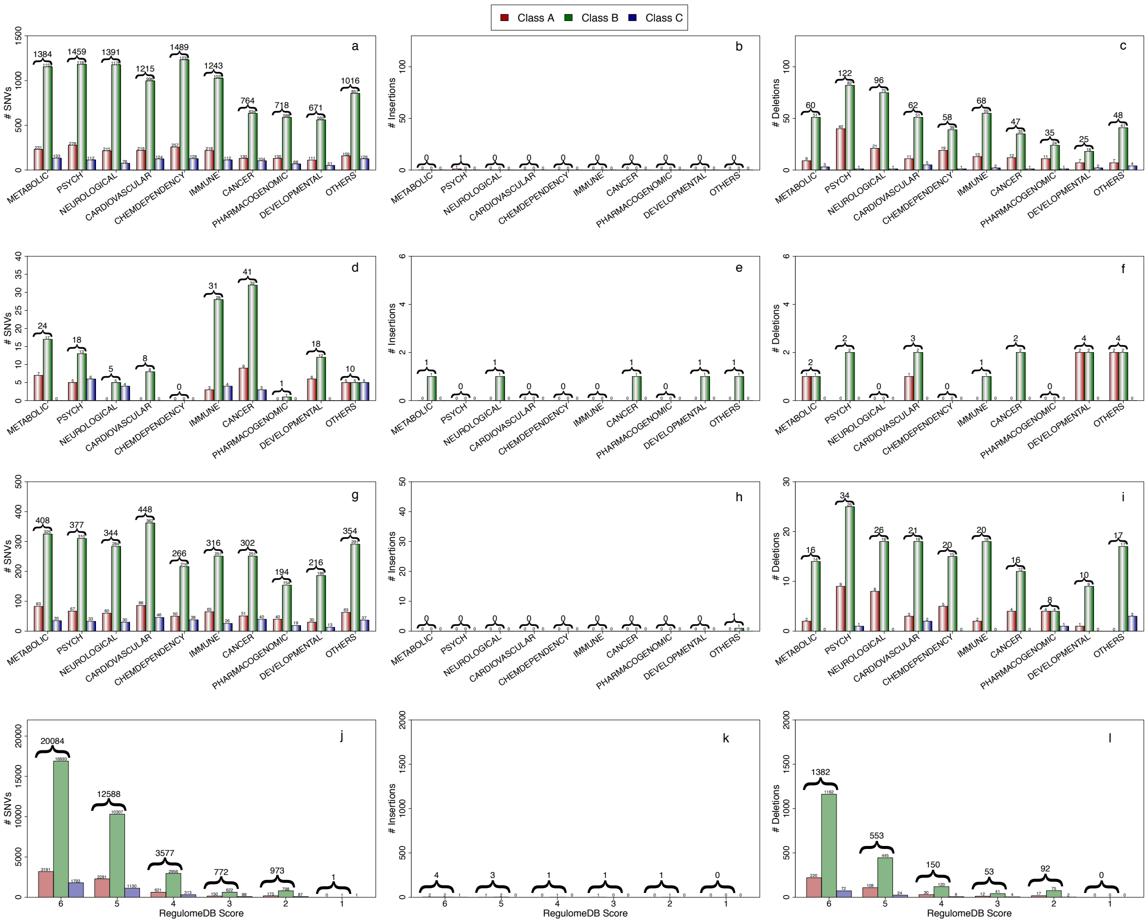

Supplemental Figure 2: GAD and RegulomeDB annotation of GRCh38 RRAs loci. Panels a, b and c report the GAD annotation of SNVs (a), Insertions (b) and Deletions (c) that belong to Protein-coding genes. Panels d, e and f report the GAD annotation of SNVs, Insertions and Deletions that belong to ncRNA biotypes. Each variant can be annotated to multiple GAD phenotype. Panels g, h and i report the GAD annotation of SNVs (g), Insertions (h) and Deletions (i) that belong to regulatory elements annotated at ENCODE. Panels j, k and l report the total number of SNVs (j), Insertions (k) and Deletions (l) as a function of RegulomeDB score. In each panel of the figure, RRA loci have been classified as belonging to class A, B and C (see main text for more details). Numbers on the top of curly brackets represent the sum of classes A and B variants.

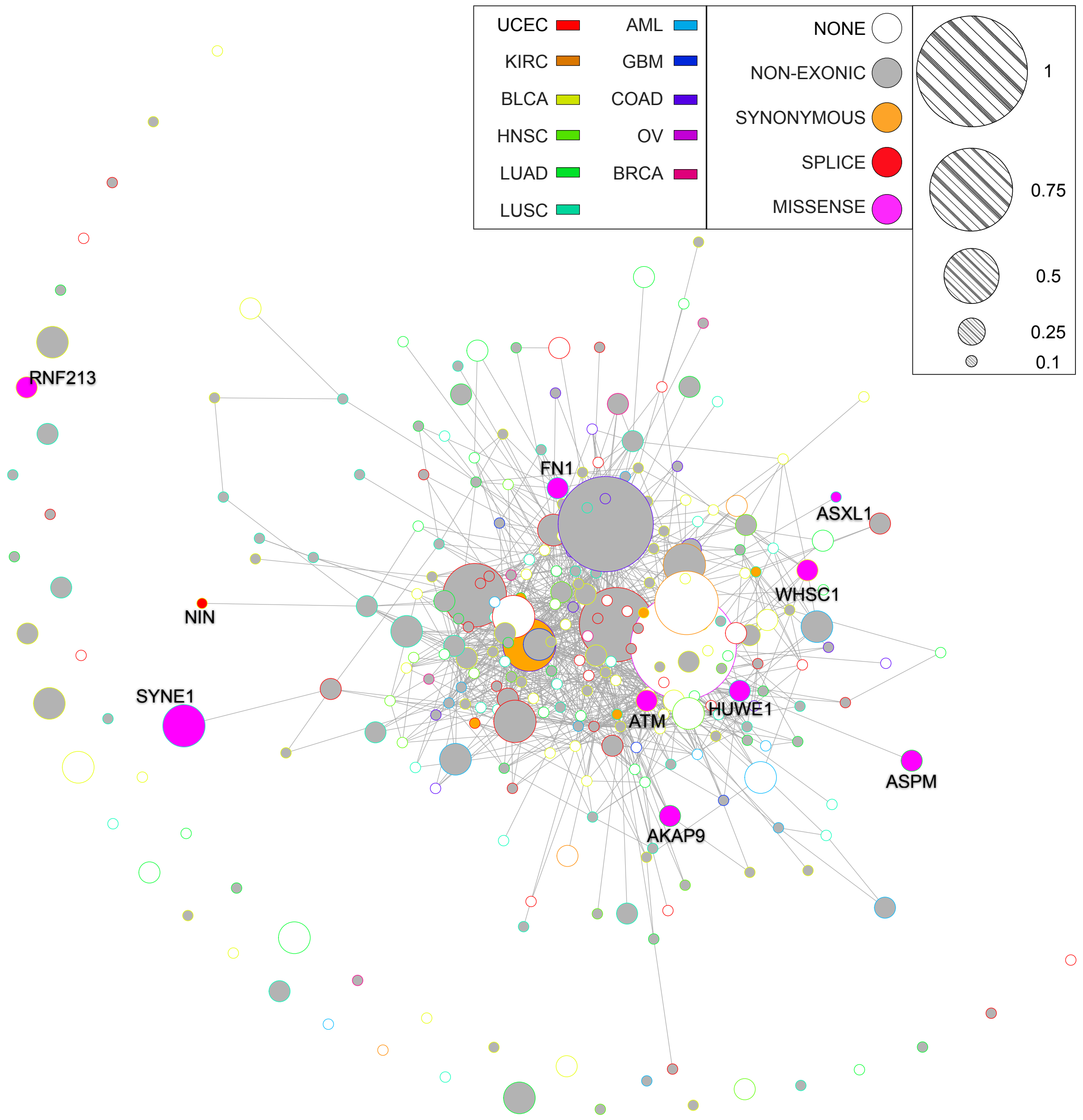

Supplemental Figure 3: Network of TCGA driver genes containing RRA loci in GRCh38. To build the network we selected all the interactions of HumanNet v1 that link the 291TCGA driver genes. Node colors represent the most severe RRA variant contained by each gene: none RRA (white), non-exonic (grey), synonymous (orange), splicing (red) and missense RRA (magenta). Node border colors represent the TCGA cancer type for which the gene has been predicted to be a driver with maximum mutation frequency: AML (Acute Myeloid Leukemia), BLCA (Bladder Urothelial Carcinoma), BRCA (Breast invasive carcinoma), COAD (Colon adenocarcinoma), GBM (Glioblastoma multiforme), HNSC (Head and Neck squamous cell carcinoma), KIRC (Kidney renal clear cell carcinoma), LUAD (Lung adenocarcinoma), LUSC (Lung squamous cell carcinoma), OV (Ovarian serous cystadenocarcinoma), UCEC (Uterine Corpus Endometrial Carcinoma), UVM (Uveal Melanoma). Node radius is a measure of the maximum mutation frequency of the driver gene in the TCGA cancer dataset coded by the border color.
